# The first enhancer in an enhancer chain safeguards subsequent enhancer-promoter contacts from a distance

**DOI:** 10.1186/s13059-019-1808-y

**Published:** 2019-09-12

**Authors:** Wei Song, Roded Sharan, Ivan Ovcharenko

**Affiliations:** 1 Computational Biology Branch, National Center for Biotechnology Information, National Library of Medicine, National Institutes of Health, Bethesda, MD USA; 2 School of Computer Science, Tel Aviv University, 69978 Tel Aviv, Israel

**Keywords:** Enhancer chain, First distal enhancer, Redundant enhancers

## Abstract

**Background:**

Robustness and evolutionary stability of gene expression in the human genome are established by an array of redundant enhancers.

**Results:**

Using Hi-C data in multiple cell lines, we report a comprehensive map of promoters and active enhancers connected by chromatin contacts, spanning 9000 enhancer chains in 4 human cell lines associated with 2600 human genes. We find that the first enhancer in a chain that directly contacts the target promoter is commonly located at a greater genomic distance from the promoter than the second enhancer in a chain, 96 kb vs. 45 kb, respectively. The first enhancer also features higher similarity to the promoter in terms of tissue specificity and higher enrichment of loop factors, suggestive of a stable primary contact with the promoter. In contrast, a chain of enhancers which connects to the target promoter through a neutral DNA segment instead of an enhancer is associated with a significant decrease in target gene expression, suggesting an important role of the first enhancer in initiating transcription using the target promoter and bridging the promoter with other regulatory elements in the locus.

**Conclusions:**

The widespread chained structure of gene enhancers in humans reveals that the primary, critical enhancer is distal, commonly located further away than other enhancers. This first, distal enhancer establishes contacts with multiple regulatory elements and safeguards a complex regulatory program of its target gene.

**Electronic supplementary material:**

The online version of this article (10.1186/s13059-019-1808-y) contains supplementary material, which is available to authorized users.

## Background

In vertebrate genomes, multiple enhancers are commonly involved in a single gene regulatory pathway by acting additively, establishing phenotypic robustness, and fine-tuning a complex gene expression pattern [1–3]. Since enhancers frequently regulate their associated genes remotely [4, 5] and sometimes skip unaffected intermediate genes [6], it is challenging to identify how a set of enhancers cooperatively regulates the same gene. In addition, the genes and their associated regulatory elements in the human genome are not uniformly distributed [7, 8], such as gene deserts which contain no protein-coding sequences but harbor multiple distant regulatory elements [8–10], making it complicated but important to explore the gene regulatory mechanisms based on a panel of multiple enhancers. Recent studies have focused on the multiple closely positioned enhancers commonly forming regions known as super-enhancers (SEs) [11–13], including the long-range enhancer interactions [14], the hierarchical structure of enhancer networks [15], and the open chromatin interactions inside SEs [16, 17].

Three-dimensional (3D) chromatin conformation experiments, such as Hi-C, provide high-resolution contact information between mapped genomic regions across human tissues and cell lines, including the associations between enhancers and their target genes [18–21]. The approach of chromatin interaction analysis by paired-end tag sequencing (ChIA-PET) provides further examples of dynamic promoter-enhancer interactions by mapping interactions between genomic regions bound by specific proteins [22]. Since the cooperative gene regulation by multiple enhancers might be related to multi-way contacts for chromatin loops [19, 23, 24], genome-wide methods such as chromosome walks (C-walks), three-way Hi-C contacts, and genome architecture mapping have been used to inform the multi-way genome aggregation of the spatial compartments in the genome [25–27]. The multi-contact 4C (MC-4C) in high resolution distinguishes cooperative from random and competing interactions to identify higher-order topological phenomena, including a group of interacting enhancers within the beta-globin SE [16]. It has also been found that the connections formed by promoters and their contacts are dynamic and tissue-specific [28, 29]. For example, during different stages of macrophage development, the activator protein-1 (AP-1)-enriched dynamic loops form a multi-loop activation cluster to control tissue-specific transcription [30]. However, current studies have not addressed (1) the organization of multiple enhancers in the 3D space and in the 1D genome, (2) the difference in genomic features between multiple interacting enhancers, (3) the biological function of a multi- enhancer regulatory program on gene expression, and (4) the genome-wide presence of multiple interacting enhancers that are not part of SEs.

To answer these questions, we performed a genomic analysis of interacting enhancers across multiple cell lines. We found that a chain of chromatin contacts may connect multiple enhancers to the same target promoter through a set of intermediate enhancers. The first enhancer in a chain with a direct contact to a promoter is often located distantly along the genome sequence but is close in the 3D space to its target gene and acts as an intermediate to bring other enhancers in an enhancer chain (EC) to their target promoters. These first enhancers maintain more Hi-C interactions with other enhancers and promoters, are more enriched for the loop factors CCCTC-binding factor (CTCF) and cohesin, and commonly overlap with the boundaries of chromatin loops. They also feature tissue specificity similar to their target promoters and preserve the primary interactions with the promoters across different cell lines, indicating their key role in gene activation and regulation. Binding sites of active transcription factors (TFs) are overrepresented in chained enhancers, and the level of gene expression associated with ECs is significantly elevated. In summary, we demonstrate that the gene regulatory programs established by a chain of multiple enhancers feature the first enhancer that directly contacts the promoter despite being positioned distantly from the promoter in the genome sequence and is essential in safeguarding gene regulation by maintaining the primary contact in the regulatory domain and bridging between the distant enhancers and their target genes.

## Results

### The primary promoter-enhancer contact is commonly established by a distal enhancer

We constructed regulatory element networks of interactions among regulatory elements in GM12878, HMEC, HUVEC, and K562 cell lines [19]. Similar to the ribonucleic acid polymerase II (RNAPII)-associated chromatin interaction network [31], all enhancers and promoters were denoted as vertices, while the significant intra-chromosome Hi-C interactions among them were denoted as edges. In total, 7374 separate networks were identified in all cell lines, and 66% of them contained at least 1 promoter. The median length of a network is 122 kb across 4 cell lines and the longest one spans 3.6 million bp (Mb) in the K562 genome. We ranked an enhancer in a regulatory element network according to its minimum number of connections to the closest promoter (i.e., E1 as a step-one enhancer and En as a step-*n* enhancer). Then, we defined an EC as a consecutive sequence of enhancers connected to a promoter (P) in the order of their ranks (P-E1-E2- … -En) and the length of an EC as the number of enhancers in that chain (e.g., an EC with a promoter and 2 enhancers has a length of 2, Additional file 1: Figure S1). Isolated promoter-enhancer contracts (EC of length 1) were not included in the set of ECs under consideration. This approach allowed us to break the network into a set of overlapping ECs. The potential influence of biases from Hi-C experiments on the ECs was evaluated (Additional file 1: Figure S2, see the “Methods” section). For an enhancer partaking in multiple ECs, an EC, in which that enhancer is at the closest rank from the promoter, was selected. Based on our definition, 9108 ECs were identified, with 2.4 enhancers per EC on average. Among 5616 promoters maintaining Hi-C contacts with enhancers, nearly half of them are associated with an EC (2626, 46%). Sixty-four percent of EC promoters are connected to multiple ECs, suggesting a genome-wide abundance of overlapping ECs. The charged multivesicular body protein 6 (CHMP6) gene involved in degrading surface receptors and in the biosynthesis of endosomes [32] is associated with the longest chain of 8 enhancers active in the GM12878 cells (Additional file 1: Figure S3A). The mitochondrial oxidase assembly protein 1 (OXA1) gene, which is related to mitochondrial adenosine triphosphate (ATP) synthase and whose mutations may cause mitochondrial encephalopathy and a combined oxidative phosphorylation defect [33], is associated with the largest number of 87 ECs (Additional file 1: Figure S3B and S3D). We applied an approach of flexible false discovery rates (FDR) to identify significant Hi-C interactions across 4 tissues, which balances the variation in the total number of ECs across different tissues (Additional file 1: Figure S3A-3C).

**Fig. 1:**
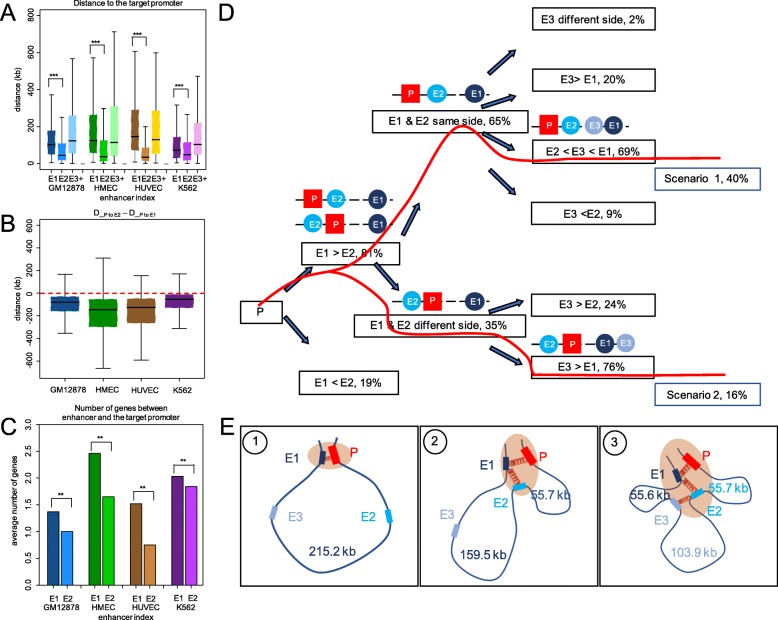
The first enhancer in an enhancer chain (EC) is distal from the target promoter (P). **a** The genomic distance between the enhancers [step-one (E1), step-two (E2), step-three, and the rest (E3+)] along the chain and their target Ps. **b** The distance between E2 and P is shorter than the distance between E1 and P. **c** E1 skips more genes to contact the target gene than E2. **d** The proportion of ECs for different cases according to the relative genomic positions of the enhancers in a chain. The red lines indicate the two most frequent organizing patterns of P, E1, E2, and, later, E3, according to their relative positions along the genome. **e** Schematic plot of a possible model for 3D organization processes of an EC in Scenario 1. **p < 0.001, ***p < 1 × 10^−10^; *p* values are calculated using the Wilcoxon rank-sum test and the binomial test

Since the position of an enhancer along an EC is the smallest number of consecutive Hi-C contacts separating this enhancer from the chain-associated promoter, the order of enhancers in an EC does not necessarily reflect their order along the sequence of the human genome. In particular, our analysis shows that for the majority of ECs, E1s are usually farther away from the promoter along the genomic sequence than E2s (Fig. 1a), independent of their upstream or downstream locations relative to the promoter (Fig. 1b). For example, across four cell lines, the median value of the distance between E1s and their target promoters is 96 kb, which is 2.1 times longer than the median distance of 45 kb for E2s (*p* value < 2.2 × 10^−16^, the Wilcoxon rank-sum test). In addition, E1s act over more genes along the sequence of the genome than E2s to search for their target promoters, further demonstrating the distal nature of the promoter-contacting enhancers, as they do not always regulate their nearest genes (Fig. 1c) [6, 28]. Our results show that the promoter recruits and directly interacts with a distal E1 instead of a proximal E2, implying unique genomic characteristics of E1s and their important role in gene regulation.

In order to explore organizing scenarios of ECs, we calculated the genomic location of each enhancer relative to the EC promoter and other enhancers in its EC. In 81% of ECs, the E1 is located farther away from the target promoter than the E2 along the genome. In addition, for E3s, 40% of them are located between the E1s and E2s and only 16% are located farther than E1s, resulting in two predominant scenarios of EC organization (Fig. 1d). Based on the most frequent scenarios, we propose a hypothesis of how an EC is built in the 3D space: (1) the E1 is distal but forms the primary chromatin contact with the target promoter and sets up a stable regulatory contact; (2) the E2, located more proximal to the promoter than the E1 along the genome, initiates a secondary interaction with the E1, bringing in additional regulatory information and stabilizing the existing regulatory contact; and (3) the following enhancers along the chain are added to the regulatory contact domain, possibly expanding the regulatory profile of the target gene. Since the alternation of the relative positions of the promoters and enhancers may lead to different 3D structures (Fig. 1d) and since the first intron of a gene has been reported to be an important component of gene regulation [34, 35], we compared cases in which EC enhancers are located upstream or downstream of promoters to investigate whether this positional preference exists in the ECs. However, we did not find a significant bias towards either of the two situations. Regardless of either the upstream or downstream position of the EC enhancers with regard to the promoters, we proposed two organization patterns for the top two most frequent situations (40% and 16% of the total cases), called scenario 1 and scenario 2 respectively in which E1 and E2 are in the same and different sides of a promoter (Fig. 1e and Additional file 1: Figure S4C).

The key reasoning behind establishing ECs was to compute the shortest distance between an enhancer and its target promoter(s) and to highlight the indirect nature of enhancer-promoter interactions in cases when an enhancer is separated from its target promoter by one or more intermediate enhancers. To further investigate the bifurcation of enhancer chains, we quantified the number of contacts between enhancers from different enhancer chains in each original regulatory network and a control set of the same network but with enhancer IDs shuffled ten times, across all four tissues. We found that, on average, about 25% of non-overlapped ECs associated with the same promoter have interactions with each other, compared to 77% in the control set, on average (*p* value < 2.2 × 10^−16^, the Wilcoxon rank-sum test). Only 18% of the EC enhancers pair with the same rank but from different ECs display inter-EC interactions, compared to 43% in the control set (*p* value < 2.2 × 10^−16^, the Wilcoxon rank-sum test) (Additional file 1: Figure S4B). These enhancer pairs also overlap both anchors of a loop with a significantly lower frequency than EC enhancer pairs (E1-E2, E2-E3, and so on), suggesting that only a few chromatin loops are formed between them (Additional file 1: Figure S4C). These relatively limited contacts between different ECs advocate for the presence of well-defined ECs and justify the selection of ECs as a backbone of our study.

The E1 performs a crucial function by connecting the promoter with the rest of the enhancers in an EC, which suggests that its function is the most competitive and essential among all enhancers in an EC. To investigate the regulatory mechanism of our proposed EC model, we next focused on the following aspects: (1) genomic characteristics of the distal E1s, (2) cooperative or competitive relationships among enhancers in an EC, and (3) the role of an E1 in an EC.

### First enhancer forms a more stable chromatin loop with the target promoter than the rest of the enhancers in an EC

**Fig. 2:**
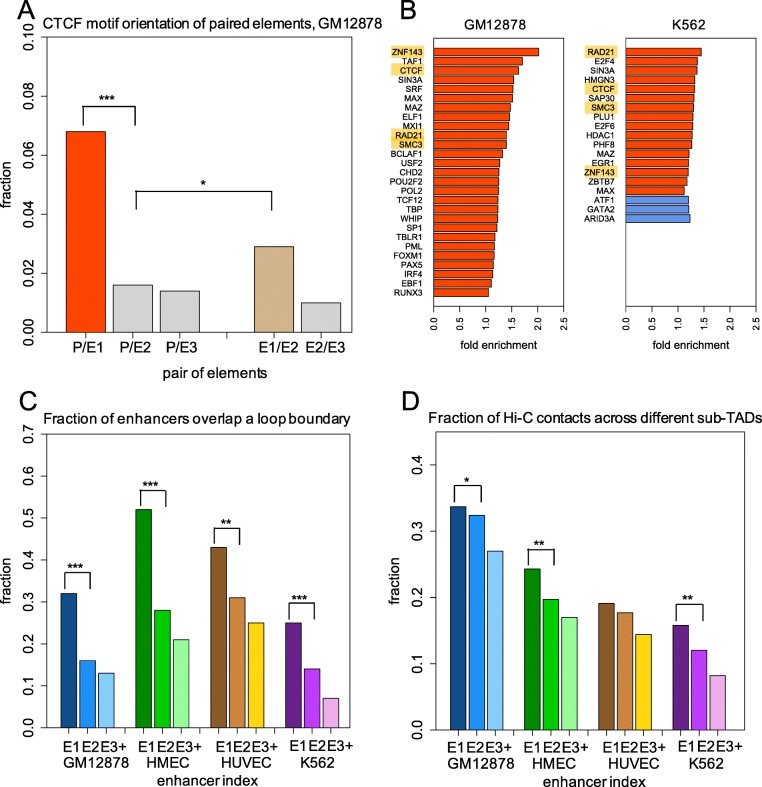
EC approach to represent a promoter-enhancer network and the preferred loop structure formed by the first enhancer in an EC with the target promoter. **a** The fraction of converged orientated CTCF motif pairs for enhancer-promoter and enhancer-enhancer loops in the GM12878 cell line. **b** The top enriched transcription factor binding sites (TFBSs) in E1s compared to E2s directly, in GM12878 and K562 cell lines. The loop factors are labeled in orange. Red and blue represent the enriched TFBSs in E1s and E2s, respectively. **c** The fraction of enhancers along the chains that overlap the loop boundaries. **d** The fraction of Hi-C contacts formed between enhancers and other enhancers/promoters in two different sub-topologically associating domains (sub-TADs). *p <  0.05, **p < 0.001, ***p < 1 × 10^−10^; *p* values are calculated using the binomial test

To reveal the distinguishing characteristics of an E1 in an EC, we started by addressing its role in a chromatin loop formation. It has been shown that the transcriptional factor CTCF mediated chromatin loops and converged orientation of the CTCF motifs near loop anchors are important for coordinated gene transcription [21]. We first compared the fraction of any two regulatory elements of an EC (for example, P-E1, P-E2, P-E3, E1-E2, and E2-E3) harboring convergent CTCF-binding sites in GM12878 cells. We observed that the CTCF motif pairs associated with E1s and their target promoters (P-E1) are more prone to convergent orientations than the pairs associated with E2s and their promoters (P-E2) (a 4.3-fold increase, *p* value < 2.5 × 10^−15^ using the binomial test). Similarly, the fraction of the converged CTCF motif pairs between E1s and E2s (E1-E2) is significantly higher than that between promoters and E2s (P-E2) (1.8-fold, *p* value < 0.005, the binomial test) (Fig. 2a). Since the convergent CTCF motifs are crucial for the formation of chromatin loops, this observation validates our hypothesis that there is no direct interaction between an E2 and a P and that their spatial contact is established through an intermediate E1. It also demonstrates a formation of two CTCF-anchored loops, one connecting P with E1 and another one connecting E1 with E2 during the formation of an indirect E2-P contact.

To further explore the characteristic features of E1s, we calculated the enrichment of transcription factor binding sites (TFBSs) in E1s using E2s as a benchmark set (using TF ChIP-seq data from GM12878 and K562 cell lines). Our results show that loop factors are the top enriched TFs in both cell lines, suggesting a DNA sequence composition of E1s predisposing them to the formation of chromatin loops by attracting specific factors (Fig. 2b). For example, in E1s of the GM12878 cell line, the density of TFBSs for CTCF and cohesin components, RAD21, and SMC3, is 1.6-, 1.4-, and 1.4-fold higher, respectively, than that in E2s (*p* value < 1.0 × 10^−4^ using the Fisher’s exact test), and a similar trend was observed in the K562 cell line. In agreement with their highly enriched loop factors, 32% of the E1s are located at the loop boundaries in the GM12878 cell line, which is 2.0-fold higher than the fraction of E2s (*p* value < 1.0 × 10^−70^, using the binomial test), indicating stable loop structures formed by them with both target promoters and the E2s (Fig. 2c). We also observed that E1s maintain a larger portion of both enhancer-promoter and enhancer-enhancer contacts across different sub-topologically associating domains (sub-TADs) than the rest of the enhancers in ECs (Fig. 2d), implying their ability to partake in distal gene regulation and to connect multiple EC enhancers to their distal target genes. However, enhancer chains largely do not cross TAD borders (98% of enhancer chains are located within a single TAD, Additional file 1: Figure S5A). Our analysis, based on the loop factors and sub-TADs, reveals an inherent ability of E1s to form stable and essential interactions with their target promoters in a gene regulatory network (GRN) through chromatin restructuring.

### First enhancers establish and maintain the essential baseline in gene regulation

**Fig. 3:**
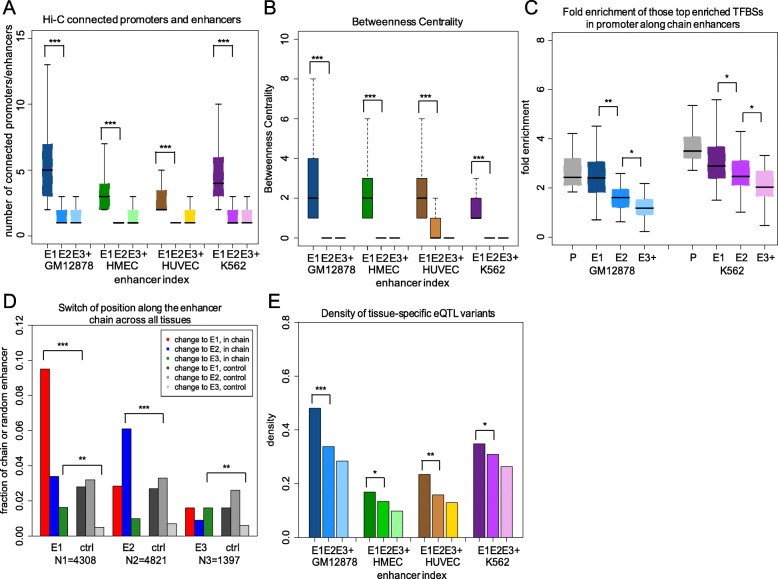
The first enhancer in an EC maintains crucial functions in gene regulation. **a** The number of Hi-C-connected promoters and enhancers for different chain enhancers. **b** The betweenness centrality (BC) scores for different enhancers. **c** The fold enrichment of the top 30 TFBSs specific to promoters regulated by ECs in GM12878 and K562 cell lines (profiled across promoters (P), E1, E2, and E3+ enhancers). **d** The fraction of EC enhancers that either maintain or switch their position in an EC across four tissues. N1, N2, and N3 are the total number of enhancers for each category of enhancers combined across four tissues. **e** The density of expression quantitative trait loci (eQTL) variants for different EC enhancers. “Whole blood” eQTLs were selected for GM12878 and K562 enhancer chains, “breast mammary” eQTLs for HMEC, and “artery aorta” eQTLs for HUVEC. *p < 0.05, **p < 0.001, ***p < 1 × 10^−10^; *p* values are calculated using the Wilcoxon rank-sum test and the binomial test

In support of the critical function of E1s in gene regulation involving multiple enhancers, we calculated the number of chromatin contacts and betweenness centrality (BC) scores for the enhancers along the same chain (see the “Methods” section). Across four cell lines, E1s feature a significantly larger number of connected promoters and enhancers than E2s (1.9-fold for average value, *p* value < 2.2 × 10^−16^ using the Wilcoxon rank-sum test). The average number of promoters and ECs connected to an E1 is 1.4 and 3.0, respectively, suggesting their direct interactions with multiple enhancers simultaneously and an ability to connect distal enhancers to the same target gene (Fig. 3a). E1s have the largest BC scores (a 5.9-fold higher average value as compared to E2s, *p* value < 2.2 × 10^−16^, the Wilcoxon rank-sum test), further supporting their central position and essential function in GRNs (Fig. 3b), which also indicates the ability of E1s to connect to multiple enhancers.

It has been found that chromatin interactions are highly dynamic and linage-specific, and the active enhancers that interact with promoters might mirror the tissue specificity of their associated genes [28]. Since an E1 is the only enhancer that directly interacts with the target promoter in an EC, its tissue specificity should be highly correlated with the tissue specificity of the target gene. To verify this, we calculated the tissue specificity similarity between the promoter and each of the enhancers along an EC. We measured their co-activities using H3K27ac peaks and computed the fraction of tissues in which each element in a pair is active (see the “Methods” section). Our results show that for cases when promoters have only one E1, E1 enhancer has an elevated tissue specificity similarity to its associated promoter compared to other EC enhancers, although this difference only reaches statistical significance for two out of four tissues (Additional file 1: Figure S5C). This finding suggests a possible role of ECs, especially E1s, in establishing the baseline tissue specificity of their target genes. However, in the case when a promoter is in contact with multiple ECs, the trend effectively disappears (Additional file 1: Figure S5D), suggesting a more complex regulatory paradigm in loci with multiple ECs regulating the same gene. Since the overall Jaccard index is low, we calculated the tissue specificity of each promoter and its associated E1 and showed that the promoters are nearly ubiquitously active across tissues and, thus, less tissue specific. By contrast, the chain enhancers, especial E1s, are very tissue-specific (Additional file 1: Figure S5E). This difference in tissue specificity between a promoter and its chain enhancers leads to a small number of common active tissues shared by them, which results in the overall small value of the Jaccard index. In addition, target promoters and E1s contain similar sets of enriched TFBSs, which might contribute to coordinated transcriptional activities and formation of a contact domain through multiple common DNA-binding proteins (Fig. 3c, Additional file 1: Figure S5B).

The observation of E1s and their target promoters being highly correlated is further confirmed by the fraction of enhancers that hold the same corresponding position in an EC across different tissues (Fig. 3d). For those chained enhancers in one tissue, E1s most likely remain as E1s (9.5%, *p* value < 10^−10^, the binomial test) instead of changing to either E2s (3.4%) or E3s in another tissue (1.6%, *p* value < 0.001, the binomial test; disregarding cases in which an enhancer does not overlap an H3K27ac mark or is not part of a chain). Similarly, E2s in one tissue tend to remain E2s (6.1%, *p* value < 10^−10^, the binomial test) again in a different tissue rather than changing to either E1s (2.8%) or E3s (1.0%). This trend reveals that E1s maintain their function in building connections with both the target promoters and E2s. The overall largest fraction of 14.5% in E1s remaining in an EC across tissues clearly suggests that they are active in more tissues and are less tissue-specific than the rest of enhancers in an EC.

To further explore the role of step-one enhancers in transcriptional events, we examined the overlap between EC enhancers and the human expression quantitative trait loci (eQTL) variants. Our result shows that the average density of tissue-specific eQTL variants is significantly higher in E1s than that in other enhancers from an EC (Fig. 3e), which validates and strengthens our previous results that E1s maintain a crucial function in gene regulation and that mutations of their sequence are likely to lead to a change in target gene expression.

### The target genes expression depends strongly on the presence and number of ECs in the locus

**Fig. 4:**
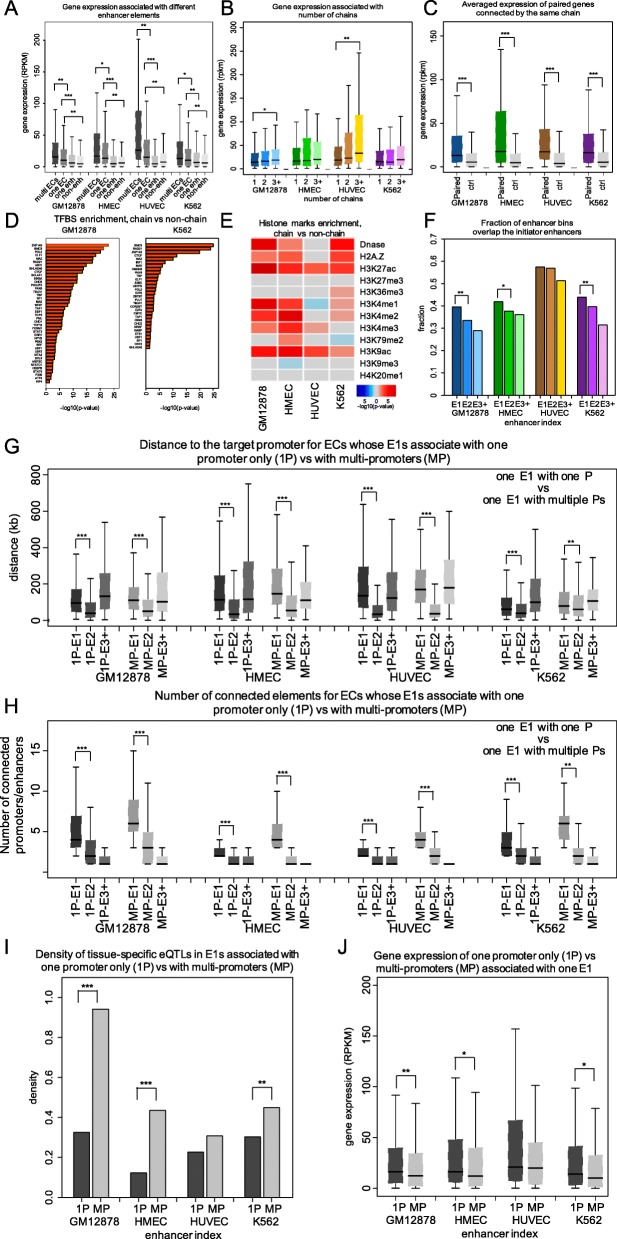
The biological functions of ECs in gene regulation. **a** The level of gene expression associated with multiple complete ECs (labeled “multi ECs”), one EC only, one enhancer in a gene locus only (one enh), and an EC connecting to the target promoter through a non-active enhancer (non-enh) DNA fragment, respectively. **b** The level of gene expression associated with different numbers of ECs. **c** The averaged gene expression for a group of genes connected by the same EC. **d** The enriched TFBSs in the EC enhancers (positive set) compared to the non-chain enhancers (control set). **e** The enrichment of histone modifications in the EC enhancers (positive) compared to the non-chain enhancers (control set). Red and blue represent the histone marks that are enriched in EC and non-chain enhancers, respectively. **f** The fraction of enhancers along the chain that overlaps the initiator enhancers. The comparison of the genomic features between ECs with E1 associated with only one promoter (1P-E1) and with multiple promoters (MP-E1) includes the distance to the target promoter (**g**), the number of connected promoters/enhancers (**h**), density of tissue-specific eQTLs (**i**), and associated gene expression levels (**j**). *p < 0.05, **p < 0.001, ***p < 1 × 10^−10^; *p* values are calculated using the Wilcoxon rank-sum test and the binomial test

We have already shown that nearly half of the target genes are associated with at least one EC and that E1s are essential for establishing a stable chromatin structure and maintaining a strong association with the target gene, making it important to investigate the functional contribution of ECs to the gene expression. First, we calculated the level of gene expression associated with ECs (Fig. 4a). We found that the presence of an EC elevates the level of gene expression significantly compared to those genes containing only one enhancer in their locus (a 1.7-fold increase, *p* value < 0.001, the Wilcoxon rank-sum test), suggesting that multiple enhancers within an EC may boost the expression of the target gene. A similar trend is observed when comparing the expression level of genes associated with multiple ECs to that of genes with one EC (a 1.9-fold increase, *p* value < 0.01, the Wilcoxon rank-sum test). There are other genomic properties that differ between E1s and single enhancers (see the “Methods” section), including the distance to the target promoter, the number of connected elements, and the overlaps with loop boundaries. Compared with single enhancers, E1 enhancers are located significantly farther away from their target promoters, they feature significantly more Hi-C contacts with promoters and other elements, and they are significantly more likely to be located at a loop boundary (Additional file 1: Figure S6A-C). This argues for fundamentally different regulatory programs established by E1 and single enhancers, suggesting that E1 enhancers are located at very specific positions in the genome which allows them to coordinate effects of other chain enhancers in the 3D chromatin space.

Second, the number of associated ECs and the level of gene expression are positively correlated, suggesting additive effects of multiple ECs on gene regulation (Fig. 4b). Third, we found that another important function of the ECs is to link separate genes and coordinate their expression. A group of genes that are connected by the same EC is more likely to be co-expressed than the random set of genes within the same distance, suggesting that different sets of regulatory information might be retrieved from the shared EC enhancers to regulate different genes (Fig. 4c). To address the value of regulatory information attributed to ECs, we compared the chain enhancers with a set of non-chain enhancers (an enhancer that does not belong to an EC) located in the same locus in respect to their enriched TFBSs (Fig. 4d). Active TFs are overrepresented specifically in chain enhancers (*p* value < 0.05, Fisher’s exact test), with an especially strong enrichment of loop factors (*p* value < 1.0 × 10^−11^, Fisher’s exact test), revealing that ECs account for a large fraction of DNA-binding regulatory events through a higher order of genomic conformations to establish a complex regulatory program of their target genes. The intensity of the epigenetic marks, including histone modifications and DNA methylation marks, which are reflective of fundamental regulatory events, was quantified next by contrasting chain and non-chain enhancers based on available ChIP-seq data (Fig. 4e). Chain enhancers display a high intensity of the H3K27ac mark of active enhancers and the H3K9ac mark of transcriptionally active genes, further supporting the crucial role of ECs in gene activation and regulation.

However, about 23% of all ECs are shared by more than one promoter, in which an enhancer is the E1 for multiple promoters. The fraction of enhancers in an EC associated with multiple promoters among all connected enhancers in the regulatory element network before the application of single ranking is 0.39, 0.13, 0.12, and 0.39 in GM12878, HMEC, HUVEC, and K562, respectively. After a single ranking, these enhancers became categorized as EC enhancers. To investigate the influence of this set of ECs on our results above, we partitioned all ECs into two separate sets: E1s associated with only one promoter (1P-E1) and E1s associated with multiple promoters (MP-E1). We compared the major genomic features between these two EC sets. The distance to the target promoter and the number of connected regulatory elements maintain similar trends between the two sets and are in agreement with the patterns for all ECs (Fig. 4g, h). Interestingly, the E1s that connect to multiple promoters have significantly more tissue-specific eQTLs than the E1s connecting to only one promoter, suggesting their critical role in regulating multiple genes (Fig. 4i). Notably, the expression levels of the genes sharing ECs are lower than those of genes with only a single EC (Fig. 4j), although the multiple genes contacting the same EC are more likely to be co-expressed (Fig. 4c), suggesting that the existence of competing target genes linked to the same EC might partially reduce the expression of each gene.

We considered two possible regulatory modes of an EC: (1) each chain enhancer has a unique, independent function, thus creating a multi-functional regulatory landscape, or (2) chain enhancers are largely redundant and all together establish a stable, but narrow, regulatory program. The first mode suggests propagation of different regulatory signals through an EC, and the second mode suggests amplification of largely the same regulatory signal along an EC. To investigate which mode is the primary mode employed by ECs, we used TFBS enrichment as a proxy of regulatory specificity and compared it among the sets of enhancers along an EC, using background DNase regions in the corresponding cell lines as the control set (Additional file 1: Figure S6D and S6E). We observed that enriched TFBSs in different chain enhancers are very similar to each other, indicating a consistency of the regulatory signal along an EC and advocating for the second, redundant regulatory mode being characteristic of ECs.

This redundancy of chain enhancers may possibly contribute to not only the fine-tuning of the target gene expression but also the coordinated expression of multiple genes connected by the same EC. Since enhancers from the same chain are highly redundant and might be involved in 3D contacts with the target promoter (Fig. 1e, Additional file 1: Figure S4C, S6D, and S6E), it is possible that any part of an EC might be sufficient for establishing and safeguarding expression of the target gene. To address this hypothesis, we focused on those genes with promoters that are connected to an EC only through a neutral DNA segment, i.e., a non-active enhancer, instead of a direct enhancer-promoter connection (Additional file 1: Figure S7A). The level of gene expression associated with these indirectly connected ECs is significantly lower than those associated with either a single enhancer or directly connected ECs, revealing an essential function of E1s in transcription and their potential role in passing regulatory signals from distal enhancers to the target promoter (Fig. 4A). In a previous study based on sequence encryption, we have identified a set of so-called initiator enhancers, which function as primary activators and intermediate catalysts of gene expression by propagating the regulatory signals of redundant enhancers to the target genes [36]. We found that initiator enhancers are significantly overrepresented among the chain enhancers as compared to the non-chain enhancers, which indicates a contribution of initiator enhancers in recruiting multiple enhancers for gene regulation through an EC structure (Additional file 1: Figure S7B). In addition, the highest enrichment of initiator enhancers was observed in E1s, which further confirms a critical function in transcriptional initiation maintained by E1s (Fig. 4f). Our results demonstrate that one possible mechanism of gene regulation by multiple enhancers is the hierarchical chain structure involving a primary contact of E1s and likely the complementary effects of redundant enhancers (Additional file 1: Figure S6D and S6E).

Finally, to explore the hypothesis that non-E1 enhancers are irrelevant to the gene regulation and simply represent open chromatin regions or false-positive enhancer predictions, we compared the density of tissue-specific eQTLs in E1, non-E1, and single and non-chain enhancers (Additional file 1: Figure S7C). The mutations in E1s have the most pronounced impact on the level of target gene expression according to the eQTL data profiled across four cell types. The eQTL density in all categories of enhancers is non-negligible, and the density in E2 and E3+ chain enhancers decreases with the degree of separation from the promoter. In addition, the fold enrichment of bound TFs according to ChIP-seq data for E1, E2, E3, and single and non-chain enhancers is at a similar level (which, in turn, is significantly higher than that in non-enhancer regions of open chromatin), suggesting that all enhancers in an EC, as well as the single and non-chain enhancers, are actively bound by TFs and contribute to the expression levels of their target genes (Additional file 1: Figure S7D).

## Discussion

Mammalian genes are commonly surrounded by multiple enhancers, a regulatory architecture that provides evolutionary stability and phenotypic robustness. Different regulatory models have been proposed to describe how these multiple enhancers coordinately regulate their target genes. However, these studies were largely focused on super-enhancers or different stages of development of the same tissue, and the general mechanisms of gene regulation by multiple regular enhancers remain unclear, including how they are organized in 3D structures and what contributions they cooperatively make to a particular gene regulatory program. We categorized active enhancers according to their 3D contacts with each other and their target promoter, dissecting the enhancer regulatory network into multiple chains of enhancers consisting of consecutive Hi-C contacts. We found that the distal E1, rather than the proximal E2 in a chain, commonly loops to the target promoter to activate the gene expression and possibly propagate the signal from other enhancers in the locus. The E2, which is usually located much closer to the promoter, loops to the E1 rather than the nearby promoter, reflecting an essential role of the E1 in coordinating the primary gene regulation, while the remaining enhancers in a chain possibly stabilize and expand an existing regulatory domain. The position of the E1 in a chain is conserved across multiple tissues, and the TFBS composition of E1s is strongly correlated with the target promoter in terms of tissue specificity and enriched TFBSs. This finding is in line with the model of gene regulation involving a static loop formed by an E1 and potential dynamic loops formed by the other enhancers in a chain [30].

Since we cannot rule out the possibility of the E2 and following enhancers (E3 to En) in a chain having direct interactions with the target promoter (albeit at a much lower frequency than the E1), it is possible that these enhancers, which are enriched for very similar but lower density of TFBSs as the E1, may partially recover the regulatory program of a gene upon a loss of the E1. However, an indirect EC connected to the target promoter through a neutral DNA segment rather than an E1 leads to a dramatic drop in the level of gene expression as compared to a directly connected chain, revealing a rather critical role of E1s in activating transcription and bridging distal enhancers to the target gene. This further confirms a hierarchical regulatory structure consisting of a primary enhancer with multiple redundant enhancers in an EC. In addition, E1s tend to connect to a single promoter and multiple (3.0 on average) ECs simultaneously. For the top 5% E1s with the most Hi-C interactions, the number of connections increases to 2.0 promoters and 12.6 ECs, suggesting their genomic functions similar to the locus control regions (LCRs) [37] and their abilities to interact with multiple enhancers equivalently. However, different from LCRs in regulating multiple genes simultaneously, E1s commonly target a single promoter only. Overall, the ability of E1s to connect multiple ECs to their target genes and the influence of ECs on gene expression indicate a possible mechanism of EC regulatory role in coordinating multiple redundant distal enhancers for target gene activation. Although our results emphasize the important and distinguishing characteristics and influence of E1s on target gene expression in contrast to other regulatory elements, such as single and non-chain enhancers, we also observe a significant contribution of E2+ enhancers, which have no direct interactions with the promoter, to the target gene expression. As part of ECs, E2+ enhancers cooperate with E1s to orchestrate gene regulation in a complex manner. It is also likely that these E2+ enhancers are stabilizing the regulatory contact domain dynamically, thereby fine-tuning and amplifying the target gene expression in a cell-specific manner.

The EC mechanism of gene regulation by multiple enhancers is very important and novel in the following aspects: (1) It indicates the crucial role and the distal feature of the first enhancer in the gene regulation. This is a very important message for identification of actual causal variants in GWAS studies, since the majority of current GWAS SNPs are found proximal to the associated promoters. Our study suggests that enhancers that are relatively far away from the promoter might act as important as the proximal enhancers. (2) We have shown that an EC is an appropriate approach to model complex GRNs involving multiple promoters and enhancers, which is also a convenient model to study the 3D organization of multiple regulatory elements. (3) A set of E1s connected to multiple promoters simultaneously features a much higher density of eQTLs than other enhancers, which might help to narrow down the search of the critical regulatory elements in certain diseases.

## Conclusions

Our study clearly demonstrates that the first but distal enhancer plays an essential role in maintaining the baseline of the target gene expression. We propose an enhancer chain model and reveal a hierarchical mechanism for gene regulation containing the first enhancer and multiple redundant enhancers. A regulatory domain is initiated from this first enhancer by building stable primary contacts with the target promoter along with a serial of redundant enhancers chained together via enhancer-enhancer interactions. In summary, our findings from this work indicate the distal and multi-contact features of the first critical enhancers and may provide insights into the organizing mechanism of complex regulatory domains.

## Methods

### Enhancer-promoter contacts and ECs

A set of genome-wide chromatin profiles of histone marks, deoxyribonuclease (DNase) I-hypersensitive sites (DHS) and transcription factor binding sites (TFBSs) was downloaded from the Encyclopedia of DNA Elements (ENCODE) and Roadmap Epigenomics projects [38, 39]. The four human cell lines with high-resolution Hi-C data [19] and gene expression data [39] were selected for this analysis: GM12878, HMEC, HUVEC, and K562 (EID: E116, E119, E122, and E123). Tissue-specific active enhancers were defined as 400 bp segments centered on H3K27ac peaks overlapping H3K4me1 peaks. Segments overlapping promoters, H3K27me3 peaks, and/or blackout regions were excluded from the list of enhancers. Promoters were defined as regions 1500 bp upstream and 500 bp downstream from a transcription start site (TSS) of a “University of California, Santa Cruz (UCSC) Known” gene [40].

We analyzed the regulatory element networks of Hi-C interactions among promoters and enhancers. Each network may contain multiple promoters and enhancers; the size of a network is defined as the genomic distance between the two most distant elements along the genome. For a selected enhancer in the network, its rank is defined as the shortest distance to its closest promoter, which is the minimum number of consecutive connections separating this enhancer from that promoter. We named the rank of enhancers as step-one (E1), step-two (E2), step-three , and the rest (E3+). An EC is defined as an oriented path from a promoter that visits enhancer nodes in the order of their ranks and should contain at least two enhancers. These chain enhancers associated with a promoter are contiguously connected through enhancer-enhancer Hi-C interactions, and only the first enhancer is connected to that promoter directly. Some examples of ECs are shown in Additional file 1: Figure S1. If a promoter is connected to only one enhancer and no other enhancers are connected to this enhancer, we call this “a single enhancer” case which is excluded from the EC category. In the case that a chain of enhancers is associated with two or more promoters, the rank of a particular enhancer will be determined by its closest promoter. We used the shortest distance approach in the construction of ECs. Namely, each enhancer was connected to the promoter using the smallest number of intermediate contacting enhancers possible. For example, in the case of a shorter and a longer path (measured as the number of contacting enhancers), only the shorter path was selected (Additional file 1: Figure S1C). In the case of two equidistant paths, both were selected (Additional file 1: Figure S1D). However, to avoid bias and double-counting, enhancers from equidistant paths were used only once in our statistical tests.

### Betweenness centrality and Jaccard index

To explore the essential enhancers in a regulatory network, we measured the centrality of each enhancer in the regulatory element network based on the number of the shortest paths between the rest of the enhancers and their target promoters which pass through this enhancer, defined as its BC score. For the cases when a promoter is connected to a single enhancer only, the BC score is set to 0. We would like to investigate if E1 is more frequently associated with underpinning the regulatory program of its target gene than the rest of the EC enhancers by calculating the tissue specificity similarity between the promoter and each of the enhancers along the chain. We measured their co-activities using H3K27ac peaks and computed the fraction of tissues in which a pair of elements is active. For example, for each pair of P-E1, we calculated the number of tissues (out of 20 total) in which both P and E1 overlap H3K27ac peaks. After that, we compared the similarity of these two lists using the Jaccard index to demonstrate the tissue specificity similarity of each P-E1 pair. Similar procedures were applied to P-E2 and P-E3 pairs.

### Hi-C data processing

Raw observed Hi-C data in 5-kb resolution, boundaries of chromatin loops, and topologically associating domains (TADs) in GM12878, HMEC, HUVEC, and K562 cell lines were retrieved from Rao et al.’s work [19]. We followed the procedure described in Huang et al.’s work to filter for significant Hi-C interactions [15]. First, we used the iterative correction and eigenvector decomposition (ICE) algorithm implemented in the Hi-Corrector package [41] to remove biases [42, 43]. After that, statistically significant interactions were identified by Fit-Hi-C [44] with the parameters “-U=2000000, -L=5000” and using a flexible false discovery rate (FDR), which is described in the following paragraph. Only *cis* (intra-chromosome) interactions are considered in this study. The ChIA-PET data of GM12878 and K562 was downloaded from GSM1872886 [21] and GSM970216, respectively.

In order to evaluate the influence of possible biases from Hi-C experiments on our results, we performed a comparison among EC enhancers to identify the potential biases caused by the fragment length and GC content in Hi-C experiments. Since the bias in distance between restriction sites and fragment length may lead to the underrepresentation of very short or very long-range interactions [45], we compared the distance between EC enhancers and the genomic regions with which they were in contact. Although E1s show a significantly larger range of contacts than other chain enhancers, the actual median values are very close among all En categories (Additional file 1: Figure S2A). The average length of interactions for E1s is only 1.1-fold higher than that for E2s. This observed difference in the range of interactions is not sufficient to explain the effects observed by this study (*p* value < 0.001, the Wilcoxon rank-sum test). One explanation for this difference might be the intrinsic ability of E1s to form distal interactions (Fig. 2c, d). In addition, the interactions for P-E1 and E1-E2 and E2-E3 are all in the middle distance, mostly shorter than 1 Mb, so the influence of fragment length should be limited for EC enhancers. Similarly, the median value of GC content is similar among all En categories in the four tissues (Additional file 1: Figure S2B). These results indicate that the Hi-C biases are not the primary factor in differentiating E1s from other chain enhancers.

To verify the possible influence of chromatin accessibility on the Hi-C interactions in our analysis, we compared the overlaps with open chromatin regions (DNase I ChIP-seq peaks) for E1 enhancers and the neutral DNA segments connecting to ECs (non-enhs). We also compared the number of elements connected through Hi-C, and the associated gene expression, as shown in Additional file 1: Figure S2. We observed a significantly higher chromatin accessibility in E1s than in non-enhs (Additional file 1: Figure S2C). However, the E1s and non-enhs have a very similar number of Hi-C-connected regulatory elements, suggesting the negligible influence of the accessibility bias in our analysis after normalization and filtering of the raw Hi-C data (Additional file 1: Figure S2D). To further demonstrate that any accessibility bias was removed from our study, we compared the associated gene expression among E1s, all non-enhs, and those non-enhs from open chromatin regions (Additional file 1: Figure S2E). We observed a marginal difference between non-enhs with different chromatin accessibility. All these results suggest that the potential bias for regions easily assayed by the Hi-C experiment was removed and did not influence our analysis.

We assumed that the promoter regions of low-expressed genes should have very few or no chromatin contacts with other parts of the genome, except for the cases of actively repressed genes regulated by connected silencers. In each cell line, the genes were defined as lowly expressed genes if their reads per kilobase million (RPKM) < 1.0 and as normal expressed genes if their RPKM ≥ 1.0 [46, 47]. The top 1000 lowly expressed genes with the highest density of H3K27me3 histone mark were assumed to be inactive genes with a small fraction of them being actively repressed. We adjusted the FDR in the Hi-C data processing, so that less than 5% of those 1000 lowly expressed genes had significant Hi-C interactions with their promoter regions. The resulting cell line-specific FDR was 2 × 10^−11^, 0.002, 0.002, and 0.0008 for GM12878, HMEC, HUVEC, and K562 cell lines, respectively. This set of FDRs not only maintains a similar distribution of length of ECs across 4 tissues (Additional file 1: Figure S3A) but also takes into account the balance between massive data in GM12878/K562 and insufficient data in HMEC/HUVEC (Additional file 1: Figure S3B and S3C), which we identified as the appropriate cutoff for identifying significant Hi-C interactions. The number of connected regulatory elements for each enhancer was normalized using a standard score (*z*-score). The top 5% enhancers with the largest *z*-scores across all networks in the corresponding cell line were selected as the group of most connected enhancers. The boundaries of chromatin loops and sub-TADs were obtained for the matching tissue in each case.

It is important to note that gene regulatory networks (GRNs) are complex and have multiple regulatory elements involved. In this analysis, we used a very stringent FDR cutoff to increase the accuracy of finding the true-positive ECs, which may have led to a reduced number of significant Hi-C interactions and associated genes as well as to the underestimation of the number of ECs. For example, out of the average 9789 genes expressed (RPKM  ≥ 1.0) across 4 cell lines, only 1404 genes feature Hi-C interactions between the promoter and at least 1 enhancer.

### Enrichment of TFBSs

For the enrichment of TFBSs in a particular set of enhancers, we took advantage of the available chromatin immunoprecipitation sequencing (ChIP-seq) TFBS data in GM12878 and K562. In each cell line, we used all the open chromatin regions marked by deoxyribonuclease I (DNase I) hypersensitivity sites (DHSs) from DNase-seq peak data as background, excluding those regions overlapping with promoters, enhancers, and blackout segments. The final control set was defined as 400-bp sequences at the centers of the remaining DNase-seq peaks. For a set of enhancers, the total number of overlaps between them and ChIP-seq peaks of a particular TFBS was summed and normalized by the total length of enhancers to get the density of the TFBS. Similarly, in the control set, the density of the same TFBS was calculated, and the ratio of these two densities was used to represent the enrichment of that TFBS in enhancers.

### Density of expression quantitative trait loci variants

The Gene-Tissue Expression (GTEx) eQTLs v7 data were obtained from the GTEx Portal (www.gtexportal.org) for the variant density analysis. For tissue-specific variants, “whole blood” eQTLs were selected for GM12878 and K562 enhancer chains, “breast mammary” eQTLs for HMEC, and “artery aorta” eQTLs for HUVEC. The density of variants was calculated as the number of variants falling into the genomic regions occupied by enhancers from a particular class over the total number of enhancers in that class.

## Supplementary information

Additional file 1: Supplementary figures. (PDF 9961 kb)

Additional file 2: Review history. (DOCX 1001 kb)

## Data Availability

The Hi-C data used in this study is from GSE63525 [19]. The ChIA-PET data of GM12878 is from GSM1872886 [21], and the ChIA-PET data of K562 is from GSM970216.
