# Supplementary material for: The first enhancer in an enhancer chain safeguards subsequent enhancer-promoter contacts from a distance
Source: Genome Biol. 2019 Sep 12;20:197. doi: 10.1186/s13059-019-1808-y (PMC6739990; doi:10.1186/s13059-019-1808-y)
Supplement: Supplementary file 1 — Supplementary figures. (PDF 9961 kb) [file 13059_2019_1808_MOESM1_ESM.pdf]

The first enhancer in an enhancer chain safeguards subsequent enhancer-promoter contacts from a distance

Additional file 1

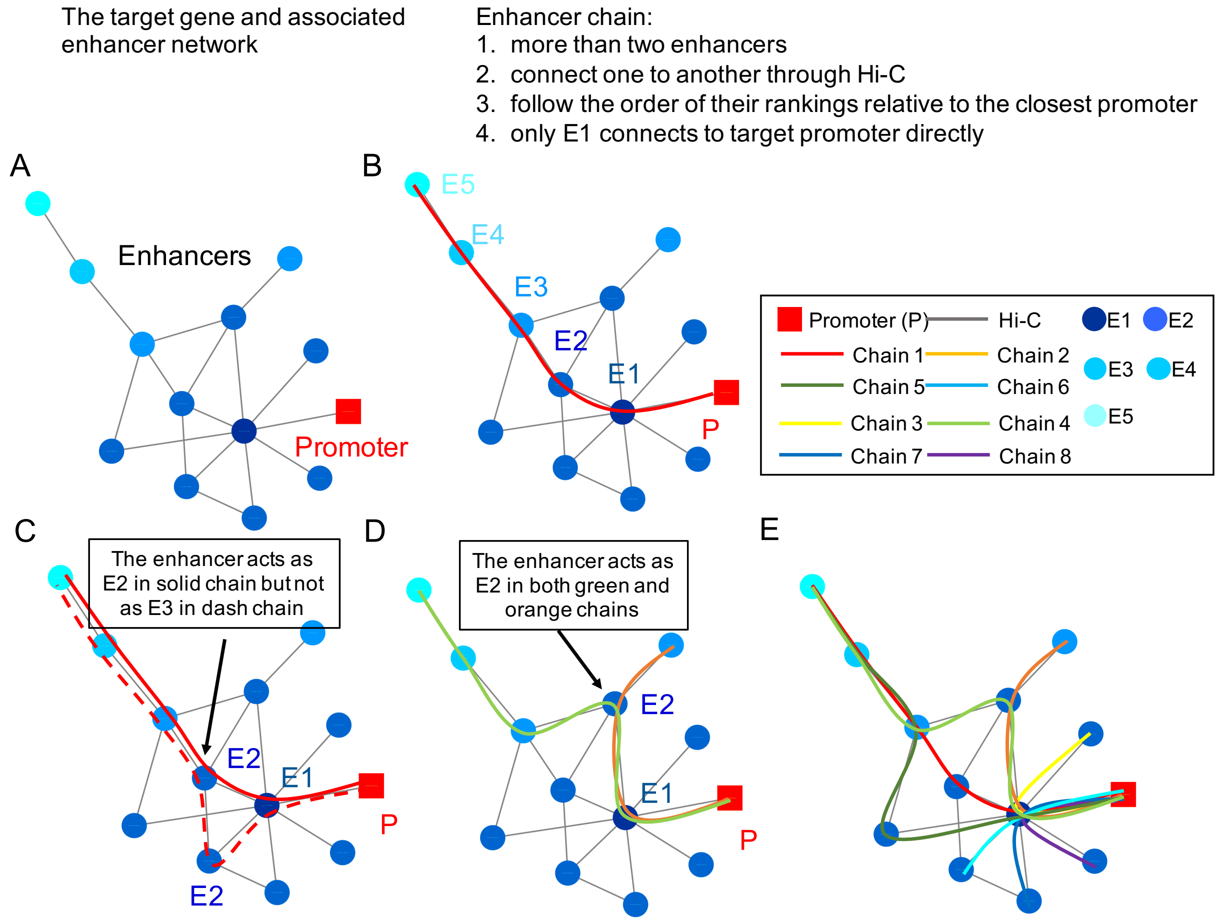

Fig. S1. Examples of enhancer chains (ECs). A) One sample of the regulatory element networks. B) An EC associated with a promoter (P) is defined as a set of contiguously connected enhancers (at least two) through enhancer-enhancer Hi-C interactions, according to their ranks associated to this promoter. The first enhancer in a chain that contacts the promoter directly is called step-one enhancer (E1), the second one in the chain is called step-two enhancer (E2), and so on. C) In the case of a shorter (solid line) and a longer (dashed line) path (measured as a number of enhancer-enhancer contacts), only the shorter path was selected. D) Different ECs may share common enhancers from the same rank. E) Theoretical schematic of enhancer chain overlaps. Eight different ECs defined according to the rank of connected enhancers relative to their closest target promoter. These ECs may share common enhancers from the same rank. At least one enhancer should be different to distinguish between two different ECs. The closest rank of an EC enhancer to the promoter is used to describe the enhancer.

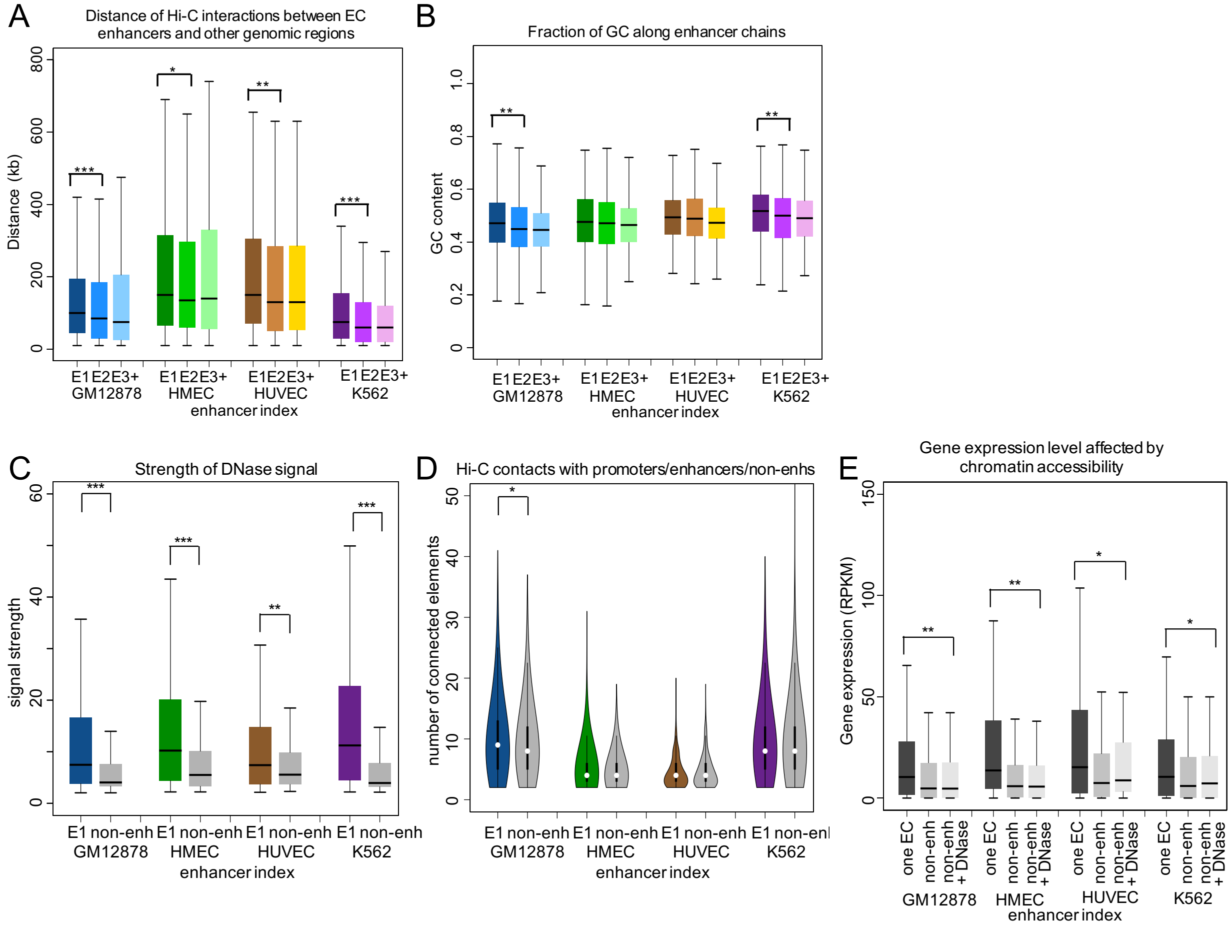

Fig. S2. Influence of the potential biases of fragment length, GC content, and an easily assayed region from Hi-C experiments. Distance of Hi-C interactions (A) and GC content (B) for chain enhancers across four tissues. C) The difference in chromatin accessibility between E1s and the neutral DNA segments connecting ECs to promoters (non-enhs). D) The number of regulatory elements connected to E1s and non-enhs. E) The level of associated gene expression for E1s, all non-enhs, and the non-enhs from open chromatin regions. * - p-value < 0.05, ** - p-value < 0.001, *** - p-value < 1×10^{-10}, p-values are calculated using the Wilcoxon rank sum test.

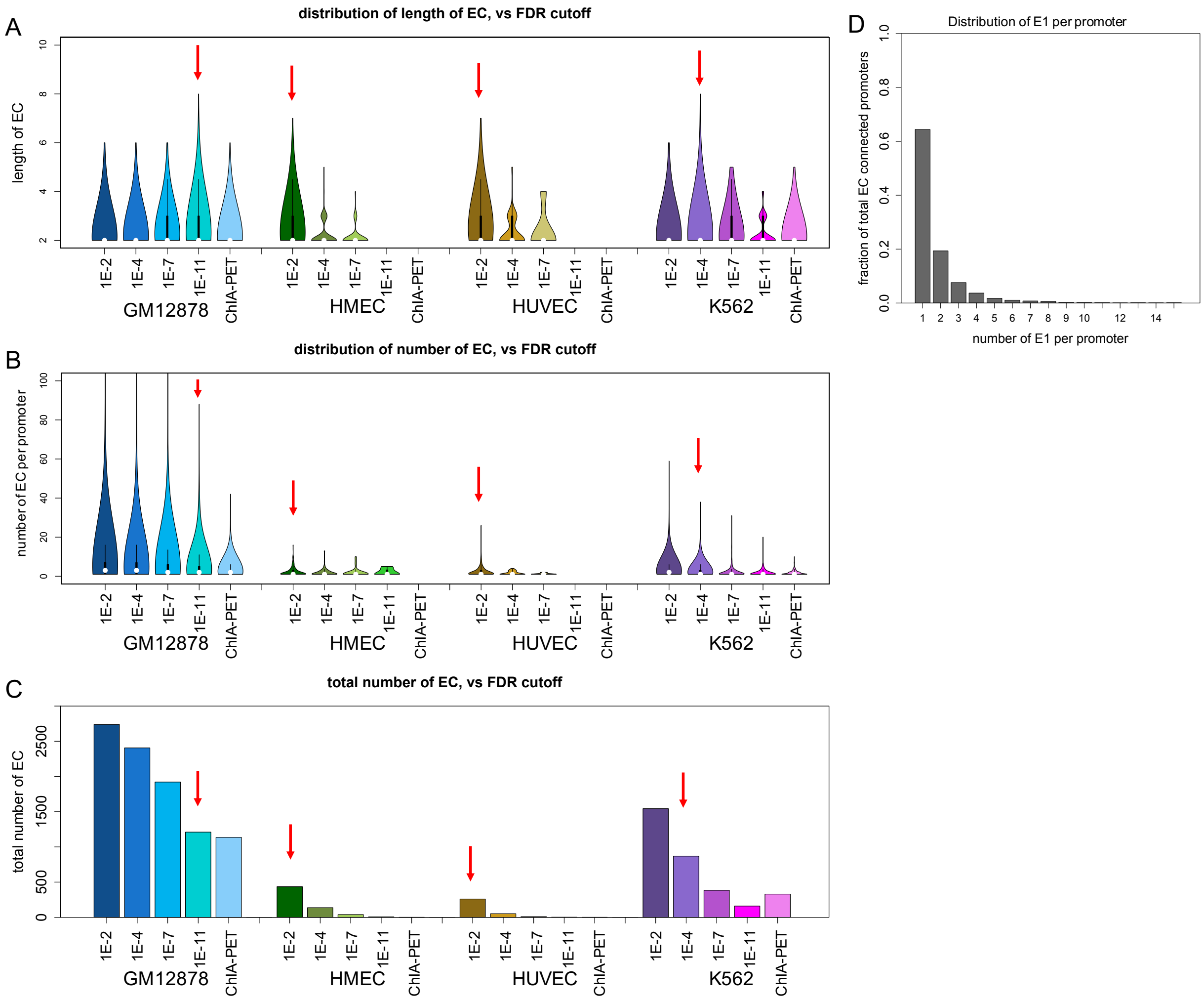

Fig. S3. The distribution of length (A) and number (B) of ECs per promoter based on different FDR cutoffs for Hi-C interactions as well as the ChIA-PET data. C) The total number of ECs vs FDR cutoffs/ChIA-PET across four tissues. The red arrows point out the selected FDRs used in this study. D) The overall distribution of E1s per promoter combined for four tissues.

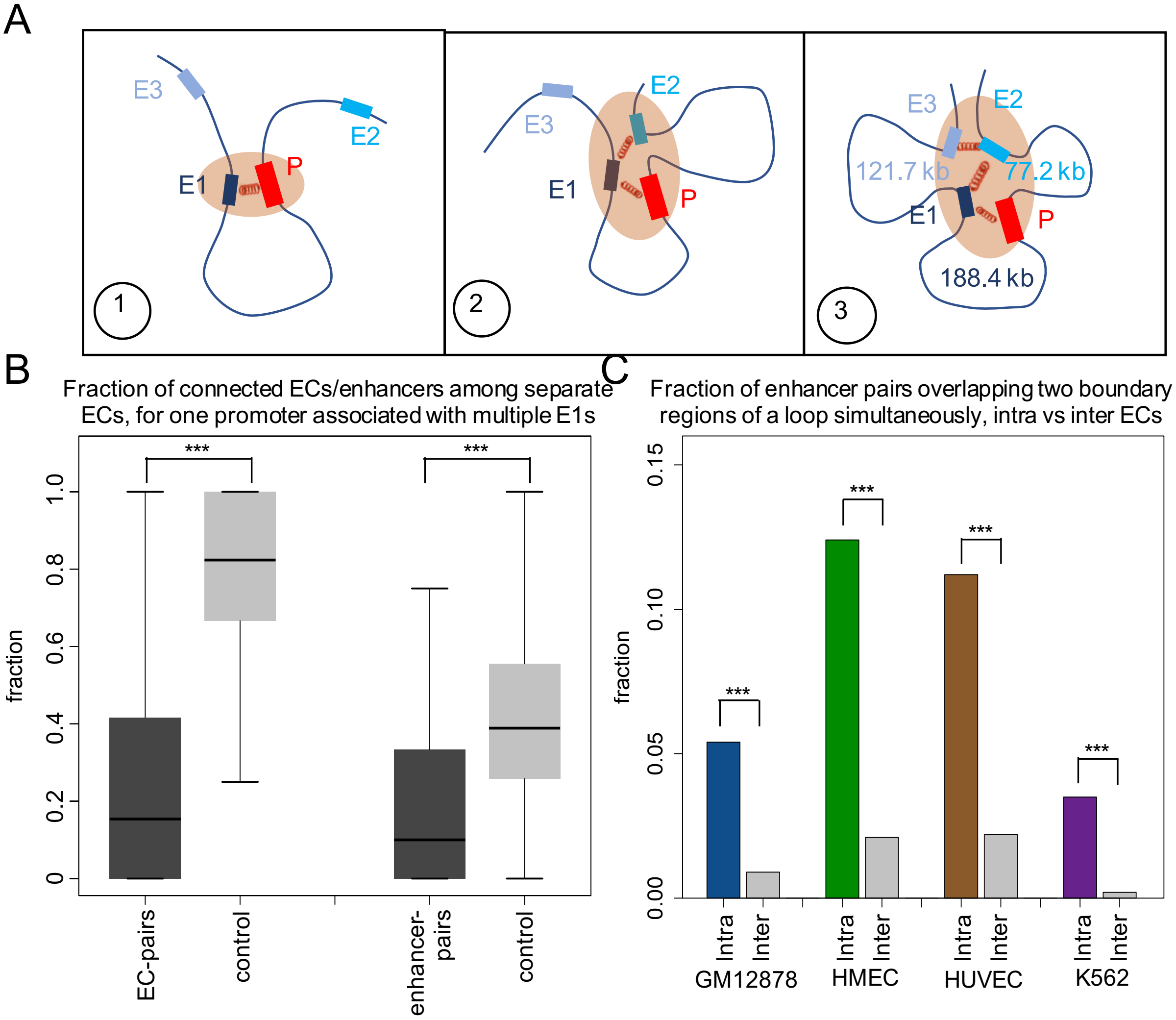

Fig. S4. A) A schematic plot of 3D contacts for the Scenario 2 EC. B) Among the non-overlapped ECs, the fraction of ECs that maintain inter-chain Hi-C interactions and the fraction of enhancer pairs from two different ECs connected by Hi-C interactions across different tissues. C) Fraction of enhancer pairs from same (intra-) and different (inter-) ECs overlap two boundaries of a loop simultaneously. *** - p-value < 1×10^{-10}, p-values are calculated using the Binomial test.

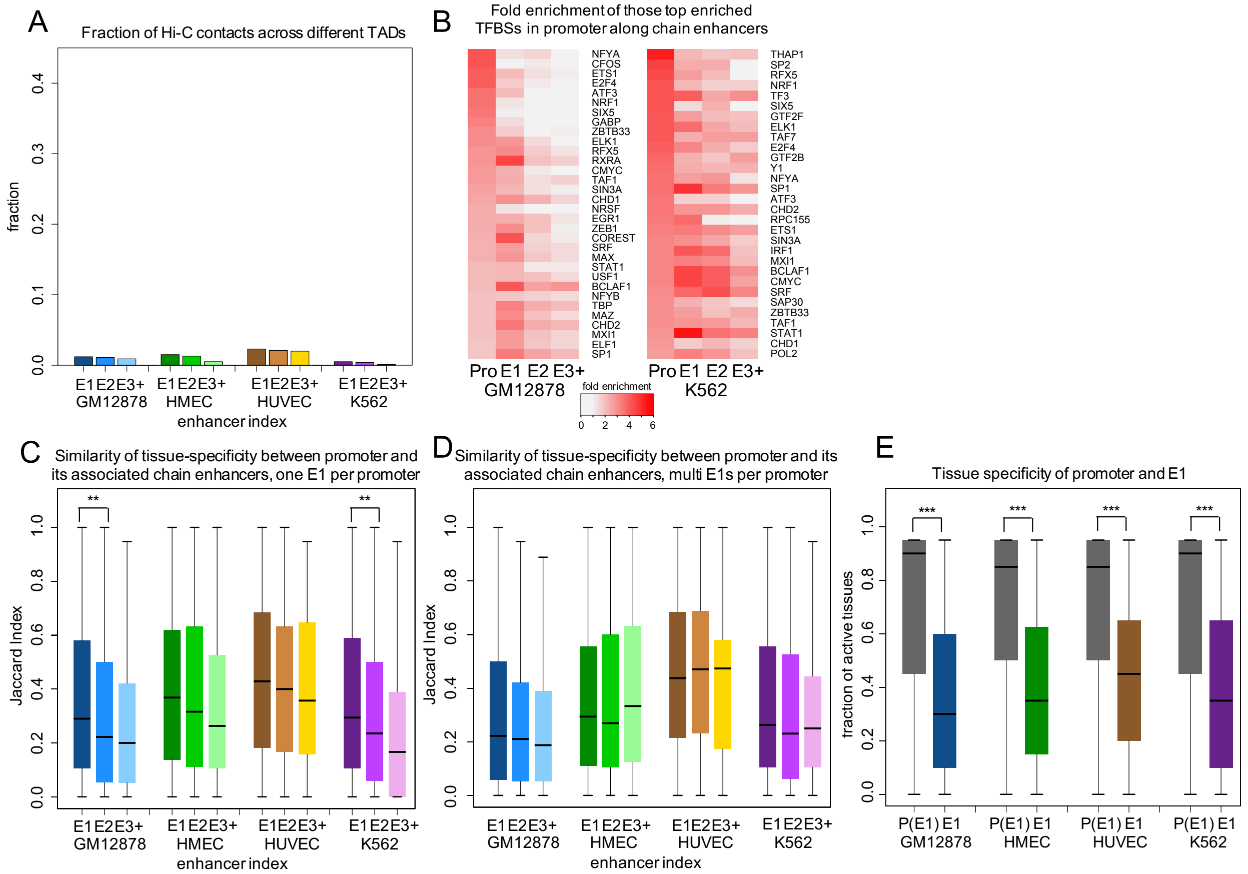

Fig. S5. A) E1 enhancers maintain the higher fraction of Hi-C interactions across sub-TADs, while EC Hi-C interactions largely remain inside individual TADs. B) The top enriched TFBSs in the target promoters and their fold enrichment in the corresponding chain enhancers for GM12878 and K562 cell lines, respectively. The tissue specificity similarity between the target promoter and associated chain enhancers, for promoters connected to a single E1 (C) and to multiple E1s (D). Jaccard Index was used to compare the enhancer and promoter lists of active tissues. E) Tissue specificity of target promoters and their associated E1s. ** - p-value < 0.001, *** - p-value < 1×10^{-10}, p-values are calculated using the Wilcoxon rank sum test.

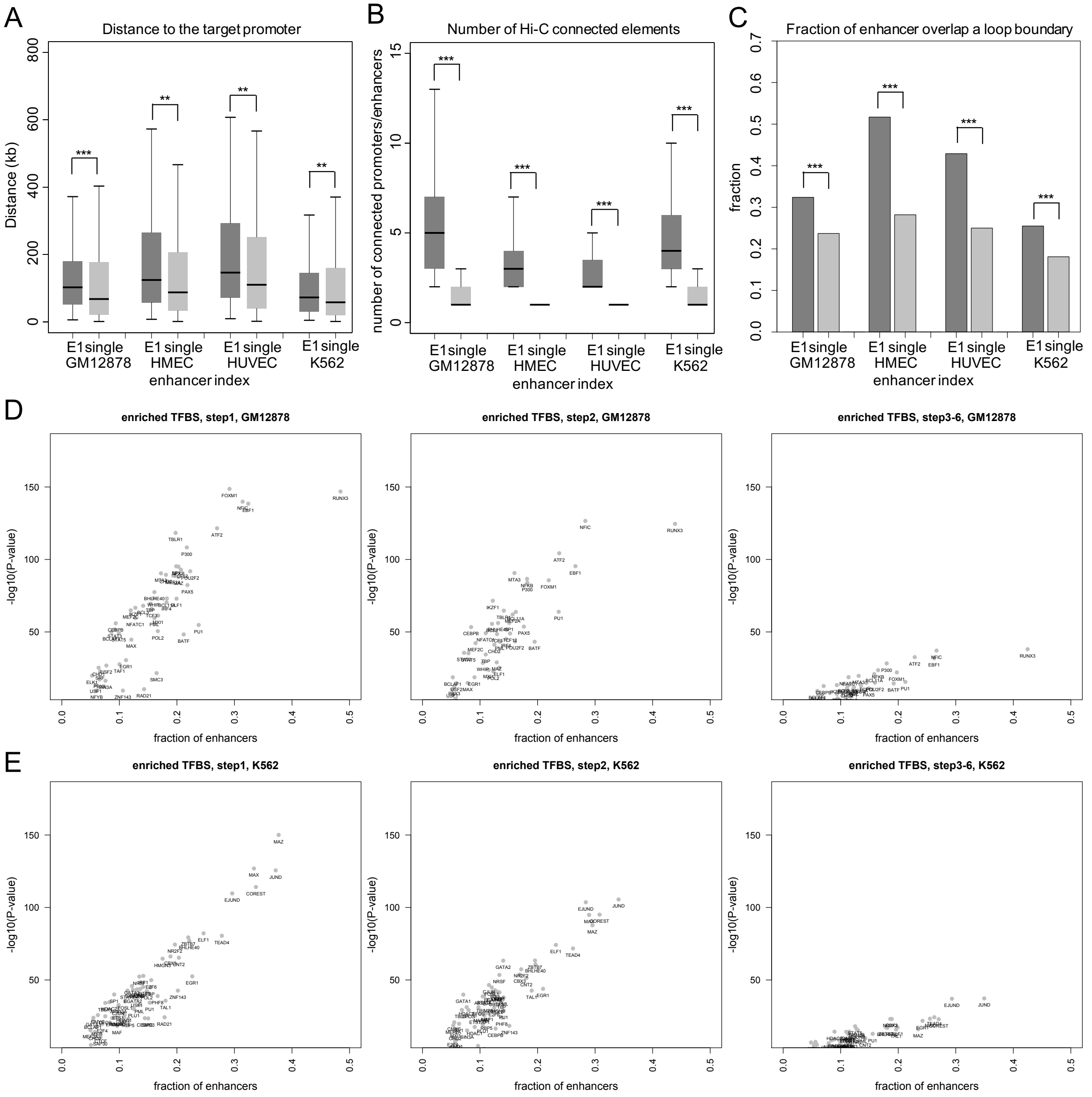

Fig. S6. Distance to the target promoter (A), number of connected promoters/enhancers (B), and overlap with loop boundaries (C) profiled for E1 and single enhancers. The enriched TFBSs in different enhancers along the chain show the redundancy of the chain enhancers, according to the overlap with enhancers and the significance of enrichment. The top and bottom panels are the GM12878 (D) and K562 (E) cell lines, respectively. P-value is calculated using Fisher's exact test. * - p-value < 0.05, ** - p-value < 0.001, *** - p-value < 1×10^{-10}, p-values are calculated using the Wilcoxon rank sum test.

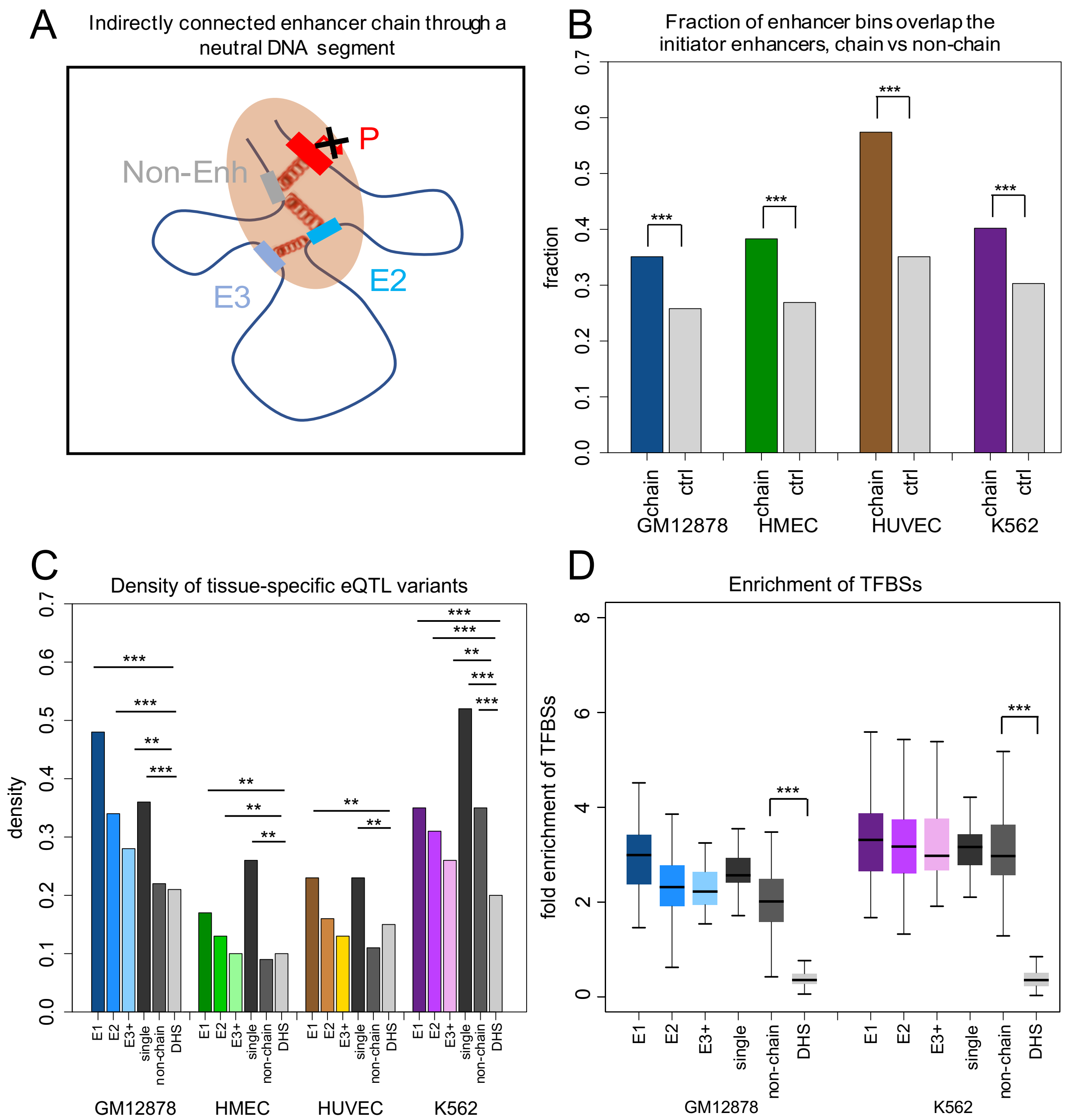

Fig. S7. A) Schematic plot of a gene associated with an EC through a non-active-enhancer (non-enh) neutral DNA segment. B) The fraction of enhancers overlapping the initiator enhancers, for chain and non-chain enhancers. C) The density of expression quantitative trait loci (eQTL) variants for EC and single and non-chain enhancers as well as non-enhancer regions of open chromatin. D) The fold enrichment of TFBSs for different enhancer categories using all open chromatin regions (DNase I ChIP-seq peaks) as control, in GM12878 and K562 cell lines, respectively. * - p-value < 0.05, ** - p-value < 0.001, *** - p-value $< 1\times10^{-10}$, p-values are calculated using the Wilcoxon rank sum test.
