# Supplementary material for: The first enhancer in an enhancer chain safeguards subsequent enhancer-promoter contacts from a distance
Source: Genome Biol. 2019 Sep 12;20:197. doi: 10.1186/s13059-019-1808-y (PMC6739990; doi:10.1186/s13059-019-1808-y)
Supplement: Supplementary file 2 — Review history. (DOCX 1001 kb) [file 13059_2019_1808_MOESM2_ESM.docx]

Review History

**First round of review**

**Reviewer 1**

**Are you able to assess all statistics in the manuscript, including the appropriateness of statistical tests used?**

yes

**Comments to author:**

The authors use Hi-C chromatin interaction data to define directed groups of interacting regulatory elements, or "enhancer chains" (ECs).  They characterize the enhancer elements found in these chains based on genomic sequence and functional attributes and suggest that the first enhancer in an EC plays a significant role in establishing stable enhancer-promoter contacts and influencing gene expression. This manuscript investigates an important and timely problem: the 3D organization and dynamics of gene regulatory elements. However, I have several comments and suggestions on the manuscript.

I found most of the network and genomic analyses reasonable and interesting, but I am concerned about the strong conclusions drawn from these results. Throughout the manuscript, the authors interpret correlations and enrichments based on existing enhancer and Hi-C maps to imply causality or the demonstration of specific functions for these elements. A few examples (out of many): 
- Line 27: It is not clear that the ECs necessarily "propagate regulatory signals" from one enhancer to another. 
- Line 141: "the following enhancers along the chain are added to the regulatory contact domain to further refine the regulatory program of a gene".  I do not believe that they have demonstrated that these enhancers further refine the regulatory program. 
- Line 336-7: "The remaining redundant enhancers are brought to the regulatory domain in a chain format, fine-tuning the target gene expression dynamically." Again, I don't think it has been proven that these necessarily are recruited in chain format or that the other enhancers are redundant or that they fine tune expression dynamically.

In general, these are reasonable hypotheses, but each would require careful, specific molecular analyses to prove. Similar claims about function are found through the manuscript and must be removed. (This are just a few examples of many.) I strongly suggest that such claims be limited to the Discussion and there clearly described as hypotheses rather than results.

I appreciated the explicit comparisons in Figure 4 of chain enhancer properties to single enhancer-gene contacts. It would be beneficial to see more of this for context in other figures.

More evidence is needed that a chain itself, which may include many enhancers that do not directly contact the target promoter, is specifically relevant to expression of the target gene. Couldn't it be that the E1s (that directly contact the promoter) are responsible for the regulation in most cases, with the enhancer-enhancer contacts (that are not near the promoter) are not functional, but merely reflect a more active open state?

I found use of the word "connectome" to describe the regulatory element contact networks (e.g., line 94) confusing. This term has been used in many contexts (most notably in neuroscience) and is thus somewhat ill defined. Furthermore, at least to me,  the "-ome" suffix suggests that the map is comprehensive and that it includes an entire set of regulatory elements. Perhaps just referring to these as regulatory chains or regulatory element networks would be more clear. 

Specific Questions:
They authors provide an example of a gene with the longest EC and the one with the highest number of ECs (n = 87). How dependent is the length and number of ECs for a promoter on the quality of the Hi-C interaction data? Are more ECs found from denser or higher quality Hi-C data? What do these distributions look like between the cell lines used?

Do biases in the regions easily assayed by Hi-C influence the results? 

Not sure about the claim that they show a more stable interaction between E1 and promoters compared to E2 and promoters (p5: 126-127).

The meaning of the red lines in Figure 1D should be clarified. Were all scenarios considered? Or does this figure indicate that only 56% of contact scenarios are analyzed?

The analysis of convergent CTCF motifs (p7: 156-165) needs to be clarified. These motifs are between the E1 and P? Shouldn't they be within or on either side of the enhancer? 

Are the loop boundaries in 2C all in reference to GM12878 (p7: 174-175)?

E1 enhancers in HUVEC cells don't contain a larger fraction of Hi-C contacts across TADs. Why this is? Also, isn't the whole point of defining TADs that there are very few interactions across their boundaries (p7: 177-180)? 

I think the number of contacts in 3A doesn't necessarily reflect the number of discrete elements that the enhancer connects to; there might be cases of many contacts to the same region or few contacts to a lot of regions that would look identical in this representation. This makes it challenging to interpret this plot.

The tissue specificity plot in 3C doesn't completely convince me that E1 having greater tissue activity similarity is a universal trend. I agree that they all have somewhat similar levels of specificity, but I find the interpretation a little strong. Also, the median Jaccard similarities are quite low. Can the authors comment on this?

In the eQTL analysis, given that many of the contexts have eQTL data from the cell line considered (or a similar tissue sample), is there tissue/cell line specificity among the enhancer chains? Does the first enhancer maintain higher density of eQTL for the relevant context?

Does the additive model take into account multiple ECs acting on the same gene?

**Reviewer 2**

**Are you able to assess all statistics in the manuscript, including the appropriateness of statistical tests used?**

There are no statistics

**Comments to author:**

The manuscript entitled "The first enhancer in an enhancer chain safeguards subsequent enhancer-promoter contacts from a distance" by Song and colleagues is an unconventional, yet interesting, approach to studying the role of 3D architecture in transcriptional regulation. The authors collect published datasets to define enhancers and promoters, the interactions between them, and the consequences on gene expression across four commonly used cell lines. They define multiple levels of regulatory units: enhancer and promoter candidates mostly by conventional methods of accessibility and histone modification ChIP signal; the connectome consisting of networks of interactions between enhancer/promoter candidates based on stringent Hi-C cutoffs; and enhancer chains (ECs) consisting of strings of consecutively ranked enhancers based on the directness of their interactions to the target promoter. 

Song et al. go on to show that many different measures for regulatory organization, activity and/or functionality display differences between enhancers within ECs that directly interact with promoters versus those that indirectly interact with promoters or are not part of ECs in the first place. While the approach taken with this study is pushing current standards in the field in a new and interesting direction, the initial assumptions are strong yet poorly motivated and explained, and inhibit a meaningful evaluation of critical aspects of the manuscript. Below is an non-comprehensive list of concerns that should be addressed in a potential revised work, which could then be properly assessed :

*     The language used in the introduction pertaining to the existence and function of enhancer hubs is too strong. Indeed, there is little direct evidence of true enhancer hubs whose activity/interactions are more than the accumulation of their individual activities/interactions.
*     The biggest concerns result from the definition of enhancer chains. 
o     First, there are several attributes of ECs that are poorly described/characterized:
♣     If an enhancer connects to multiple ECs and is chosen for one based on its closest rank, are the other connections completely ignored, and/or does this affect the rank of this enhancer relative to the non-chosen ECs? 
♣     How are chains defined if an enhancer is the E1 for multiple promoters? 
♣     How often are enhancers part of ECs with multiple promoters before rank-based choices?
♣     Lines 107-108. What is the distribution of the number of E1s per promoter?
♣     How do you define a distinct EC? Is it different E1s, or any different combination of participating enhancers or any different ordering? Given the example EC in Supplemental Fig. 1A, one can draw many different paths that connect consecutive enhancer ranks. Are these all considered different ECs?
o     Secondly, is there any reason to believe that a chain-based, single path analysis approach is the correct or preferable approach? This is especially important in cases where a given promoter has multiple direct "E1" contacts. What is the motivation to consider chains as separate units?
*     Why look at the statistics of enhancers being upstream or downstream of promoters? Is there any evidence that this relative positioning in the linear genome is somehow important?
*     Lines 136-141. These are hypotheses, quite far from evidence-based facts, and should therefore be stated as such.
*     Is Fig 2a not mostly the result of your definition of E2s, which do not have evidence of direct contact with the promoter and therefore shouldn't show convergent ctcf motifs?
*     Are E1s different in terms of fragment length and/or GC content? Presumably these known technical artifacts for Hi-C would be normalized out with ICE, but this is a necessary sanity check.
*     Line 189, how can the average number of promoter-E1 contacts be less than 1? I thought an enhancer could only be an E1 if it directly contacts a promoter?
*     Line 191, how can E1s have such large BC scores when in the methods it states that E1 BC scores are set to zero?
*     Line 194. Maybe this suggests that E1s are *constituents* of hub enhancers, but, as I understand your definition, an E1 is one enhancer and therefore can't be a hub by itself.
*     Line 198-199, This is only worth considering if the promoter has only one E1, otherwise it is misleading?
*     Exactly what is being measured for the Jaccard index in figure 3c?
*     It is not so clear how much more similar E1 is to promoters compared to others (Figure 3d). It seems what is presented is not actually a direct comparison between the two.
*     Figure 3e, these are very low numbers, and it is unclear that they mean anything.
*     Line 233-236, this does not indicate an additive function.
*     Figure 4. It seems possible that single and/or non-chain enhancers used for comparisons in this figure could just be false-positive enhancer candidates. Is there any strong evidence to consider these single or non-chains regions as enhancers at all? This question could also be posed for E1 versus E2,3,4… enhancers. Could it be that E1 enhancers are true enhancers and the others just be false positives?


Once the authors provide a sufficiently detailed characterization of their EC units according to the points above and provide reasonable answers to most of these questions, the study can be properly evaluated in terms of its results regarding characteristics of 3D architecture related to transcriptional regulation. As is, though, the EC starting definition is too vague and has too many potential caveats.

**Authors Response**

**Point-by-point responses to the reviewers’ comments:**

Reviewer #1: I found most of the network and genomic analyses reasonable and interesting, but I am concerned about the strong conclusions drawn from these results. Throughout the manuscript, the authors interpret correlations and enrichments based on existing enhancer and Hi-C maps to imply causality or the demonstration of specific functions for these elements. A few examples (out of many):

1. - Line 27: It is not clear that the ECs necessarily "propagate regulatory signals" from one enhancer to another.

*Following the reviewer’s suggestion and to avoid overstatement, we changed the corresponding sentence to read as “…, suggesting an important role of the first enhancer in initiating transcription of the target promoter and bridging the promoter with other regulatory elements in the locus.”*

2. - Line 141: "the following enhancers along the chain are added to the regulatory contact domain to further refine the regulatory program of a gene". I do not believe that they have demonstrated that these enhancers further refine the regulatory program.

*We made sure that the statement on the role of these secondary enhancers in refining the regulatory program of the target gene is presented as a hypothesis and not a validated result, and the revised sentence reads as “…the following enhancers along the chain are added to the regulatory contact domain, possibly expanding the regulatory profile of the target gene.”*

3. - Line 336-7: "The remaining redundant enhancers are brought to the regulatory domain in a chain format, fine-tuning the target gene expression dynamically." Again, I don't think it has been proven that these necessarily are recruited in chain format or that the other enhancers are redundant or that they fine tune expression dynamically.

*By explicitly citing the Supplemental Figure S7 results in the revised version of the manuscript, we are clarifying that the redundancy is limited to the redundancy in tissue-specificity previously observed by this study, and we avoid stating that the chaining is a result of a “supervised” recruitment of enhancer (which we couldn’t prove), stating that it is one of the observations: “… along with a serial of redundant enhancers (Supplemental Figure S7D and S7E) chained together via enhancer-enhancer interactions…”*

*In addition, we performed an additional analysis to demonstrate that the chain format is an appropriate approach to model the complex promoter-enhancer regulatory networks (Supplemental Figure S4B and S4C, please also see our response to the second reviewer’s question #7)*

4. In general, these are reasonable hypotheses, but each would require careful, specific molecular analyses to prove. Similar claims about function are found through the manuscript and must be removed. (This are just a few examples of many.) I strongly suggest that such claims be limited to the Discussion and there clearly described as hypotheses rather than results.

*We completely agree with the reviewer’s opinion and apologize for the overstatements presented in the original version of the manuscript. We carefully edited the manuscript to make sure the hypotheses are clearly separated from results. Several statements were moved to the Discussion, and these statements are now presented as hypotheses.*

*There are two specific examples of the changes made to the manuscript (in addition to the three changes detailed above):*

*1). We changed the sentence: “… ECs coordinately organize multiple redundant distal enhancers for fine-tuning and amplifying the expression of their target genes” to “… a possible mechanism of EC regulatory role in coordinating multiple redundant distal enhancers for target gene activation”*

*2). We changed the sentence: “… the hierarchical chain structure involving a primary contact of E1s and the complementary effects of redundant enhancers for fine-tuning and stabilizing the target gene expression” to “… the hierarchical chain structure involving a primary contact of E1s and likely complementary effects of redundant enhancers (Supplemental Figure S7D and S7E) for fine-tuning and stabilizing the target gene expression”*

*3). We moved the hypothesis about redundant chain enhancers to the end of the Discussion section, and it now reads as: “These redundant enhancers are possibly stabilizing the regulatory contact domain dynamically, fine-tuning and amplifying the target gene expression.”*

*Other changes are highlighted in red in the revised version of the manuscript.*

5. I appreciated the explicit comparisons in Figure 4 of chain enhancer properties to single enhancer-gene contacts. It would be beneficial to see more of this for context in other figures.

*As suggested by the reviewer, we performed additional analyses focusing on the comparison between E1 and single enhancers, including distance to the target promoter, number of Hi-C interactions, and overlap with loop boundaries. These results suggest that E1 enhancers are more distal to the target promoter as compared to single enhancers. E1 enhancers connect to significantly more promoters and other enhancers, and form loops with target elements more frequently than single enhancers, further emphasizing a functional difference between these two classes of enhancers. These results are summarized in the Results section and Supplemental Figure S7: “There are other genomic properties that differ between E1 and single enhancers, including the distance to the target promoter, the number of connected elements, and overlaps with loop boundaries. Compared with the single enhancers, E1 enhancers are located significantly farther away from their target promoters, they feature significantly more Hi-C contacts with promoters and other elements, and they are significantly more likely to be located at a loop boundary (Supplemental Figures S7A-C). This argues for fundamentally different regulatory programs established by E1 and single enhancers, suggesting that E1 enhancers locate at very specific positions in the genome that allow them to coordinate effects of other chain enhancers in 3D chromatin space.”*

*Supplemental Figure S7. Distance to the target promoter (A), number of connected promoters/enhancers (B) and overlap with loop boundaries (C) profiled for E1 and single enhancers.*

6. More evidence is needed that a chain itself, which may include many enhancers that do not directly contact the target promoter, is specifically relevant to expression of the target gene. Couldn't it be that the E1s (that directly contact the promoter) are responsible for the regulation in most cases, with the enhancer-enhancer contacts (that are not near the promoter) are not functional, but merely reflect a more active open state?

*To explore the hypothesis that non-E1 chained enhancers are irrelevant to gene regulation and simply represent open chromatin regions, we quantified the density of tissue-specific eQTLs in E1 and non-E1 enhancers (Supplemental Figure S8; copied below). While the mutations in E1 enhancers have the most pronounced impact on the level of target gene expression according to the eQTL data profiled across four cell types, the eQTL density in E2 and E3+ chain enhancers is non-negligible and decreases with the degree of separation from P (Supplemental Figure S8C). In addition, the fold-enrichment of bound TFs according to ChIP-seq data for E1, E2, and E3 is at a similar level (which, in turn, is significantly higher than in non-enhancer regions of open chromatin), suggesting that all enhancers in an EC are actively bound by TFs and contribute to the expression levels of their target genes (Supplemental Figure S8D). We added these new results to the Results section 4 of the revised manuscirpt: “Finally, to explore the hypothesis that non-E1 enhancers are irrelevant to gene regulation and simply represent open chromatin regions or false positive enhancer predictions, we compared the density of tissue-specific eQTLs in E1, non-E1, and single and non-chain enhancers (Supplemental Figure S8). The mutations in E1s have the most pronounced impact on the level of target gene expression according to the eQTL data profiled across four cell types. The eQTL density in all categories of enhancers is non-negligible, and the density in E2 and E3+ chain enhancers decreases with the degree of separation from the promoter. In addition, the fold enrichment of bound TFs according to ChIP-seq data for E1, E2, E3, and single and non-chain enhancers is at a similar level (which, in turn, is significantly higher than that in non-enhancer regions of open chromatin), suggesting that all enhancers in an EC, as well as the single and nonchain enhancers, are actively bound by TFs and contribute to the expression levels of their target genes (Supplemental Figure S8C and S8D).”*

*Figure S8. The density of expression quantitative trait loci (eQTL) variants for EC and single and non-chain enhancers as well as non-enhancer regions of open chromatin (DHS). The fold enrichment of TFBSs for different enhancer categories using all open chromatin regions (DNase I ChIP-seq peaks) as control, in GM12878 and K562 cell lines, respectively.*

7. I found use of the word "connectome" to describe the regulatory element contact networks (e.g., line 94) confusing. This term has been used in many contexts (most notably in neuroscience) and is thus somewhat ill defined. Furthermore, at least to me, the "-ome" suffix suggests that the map is comprehensive and that it includes an entire set of regulatory elements. Perhaps just referring to these as regulatory chains or regulatory element networks would be more clear.

*We agree with the Reviewer. Following this suggestion, the term “connectome” was changed to “regulatory element networks” in the revised version of the manuscript.*

Specific Questions:

8. They authors provide an example of a gene with the longest EC and the one with the highest number of ECs (n = 87). How dependent is the length and number of ECs for a promoter on the quality of the Hi-C interaction data? Are more ECs found from denser or higher quality Hi-C data? What do these distributions look like between the cell lines used?

*To demonstrate the correlation between the EC and the FDR cutoff for Hi-C interactions, we calculated the distribution of length and number of ECs per promoter according to different FDR cutoffs (1E-2, 1E-4, 1E-7, 1E-11) used in four corresponding tissues, as well as the available CTCF-mediated ChIA-PET data for GM12878 and K562 (downloaded from GSM970216 and GSM1872886, Supplemental Figure S3). As shown in Supplemental Figure S3A, for the length of EC per promoter, the decreasing of FDR value has little impact on GM12878 and K562, while smaller FDRs dramatically decrease the length of EC in HMEC and HUVEC. However, as shown in Supplemental Figure S3B and S3C, the number of ECs per promoter and the total number of ECs all decrease as the FDR cutoff becomes smaller, suggesting that more ECs found from denser Hi-C data. Comparison across four tissues also suggests the systematic divergence in HiC data and the downstream EC analysis.*

*We tried to reduce this influence from the quality issue across tissues by using adjustable FDRs in Hi-C data processing. Specifically, we used the same criteria to identify significant Hi-C interactions, which is only 5% of most lowly expressed genes maintaining Hi-C interactions in their promoter regions for the corresponding tissue. The resulting FDR cutoffs we used in analysis were 2x10-11, 0.002, 0.002 and 0.0008 for GM12878, HMEC, HUVEC, and K562 cell lines, respectively (shown in red arrows in the plot). This set of FDRs not only maintain similar distribution of length of EC across four tissues (Supplemental Figure S3A), but also take into account the balancing between massive data in GM12878/K562 and insufficient data in HMEC/HUVEC (Supplemental Figure S3B and S3C), which we think are appropriate cutoffs for finding significant Hi-C interactions.*

*We added a clarification statement in the Results section 1, Methods section and Supplemental Figure S3:*

*Statement in Results section 1: “We applied an approach of flexible false discover rates (FDR) to identify significant Hi-C interactions across four tissues, which better reduces the variation in the total number of ECs across different tissues (Supplemental Figure S3A-3C).”*

*Statement in Methods section 3: “This set of FDRs not only maintains a similar distribution of length of ECs across four tissues (Supplemental Figure S3A), but also takes into account the balance between massive data in GM12878/K562 and insufficient data in HMEC/HUVEC (Supplemental Figure S3B and S3C), which we identified as the appropriate cutoffs for identifying significant Hi-C interactions.”*

*Supplemental Figure S3. The distribution of length (A) and number (B) of ECs per promoter based on different FDR cutoffs for Hi-C interactions as well as the ChIA-PET data. C) The total number of ECs vs FDR cutoffs/ChIA-PET across four tissues. The red arrows point to the selected FDRs used in this study.*

9. Do biases in the regions easily assayed by Hi-C influence the results?

*A: In this work, in addition to applying the ICE algorithm developed for leveraging the unique pairwise and genome-wide structure of Hi-C data to achieve equal visibility across all genomic regions [Imakaev, M. et. al. Nat. Methods, 2012, 9:999], the Hi-Corrector package was also used to account for the memory-efficient normalization of high resolution (5 kb) Hi-C data with ICE implemented. A bias parameter per bin/locus was calculated, so that bias = 1 (expected amount of count/visibility), bias > 1 (higher than expected count), bias < 1 (lower than expected count). This bias information was subsequently used by Fit-Hi-C to further identify statistically significant interactions.*

*To verify the possible influence of chromatin accessibility on the Hi-C interactions in our analysis, we compared overlaps with open chromatin regions (DNase I ChIP-seq peaks) for E1 enhancers and the neutral DNA segments connecting to ECs (non-enhs). We also compared the number of elements connected through Hi-C, and the associated gene expression, as shown in Supplemental Figure S2. We observed a significantly higher chromatin accessibility in E1s than in non-enhs (Supplemental Figure S2C). However, the E1s and non-enhs have a very similar number of Hi-C-connected regulatory elements, suggesting the negligible influence of the accessibility bias in our analysis after normalization and filtering of the raw Hi-C data (Supplemental Figure S2D). To further demonstrate that any accessibility bias was removed from our study, we compared associated gene expression among E1s, all non-enhs, and those nonenhs overlapping with DNase signals (Supplemental Figure S2E). We observed a marginal difference between non-enhs with different chromatin accessibility. All these results suggest that the potential bias for regions easily assayed by the Hi-C experiment was removed and did not influence our analysis.*

*We added this information to the Results section 3 and Supplemental Figure S2: “To verify the possible influence of chromatin accessibility on the Hi-C interactions in our analysis, we compared overlaps with open chromatin regions (DNase I ChIP-seq peaks) for E1 enhancers and the neutral DNA segments connecting to ECs (non-enhs). We also compared the number of elements connected through Hi-C, and the associated gene expression, as shown in Supplemental Figure S2. We observed a significantly higher chromatin accessibility in E1s than in non-enhs (Supplemental Figure S2C). However, the E1s and non-enhs have a very similar number of Hi-C-connected regulatory elements, suggesting the negligible influence of the accessibility bias in our analysis after normalization and filtering of the raw Hi-C data (Supplemental Figure S2D). To further demonstrate that any accessibility bias was removed from our study, we compared associated gene expression among E1s, all non-enhs and those nonenhs from open chromatin regions (Supplemental Figure S2E). We observed a marginal difference between non-enhs with different chromatin accessibility. All these results suggest that the potential bias for regions easily assayed by the Hi-C experiment was removed and did not influence our analysis.”*

*Supplemental Figure S2. C) The difference in chromatin accessibility between E1s and the neutral DNA segments connecting ECs to promoters (non-enhs). D) The number of regulatory elements connected to E1s and non-enhs. E) The level of associated gene expression for E1s, all nonenhs, and the non-enhs from open chromatin regions.*

10. Not sure about the claim that they show a more stable interaction between E1 and promoters compared to E2 and promoters (p5: 126-127).

*To clarify and avoid a speculation about the stability of the promoter-E1 interaction, we changed our statement from “Our results show that promoters form a stable interaction with distal E1s instead of proximal E2s, implying …” to “Our results show that the promoter recruits and directly interacts with a distal E1 instead of a proximal E2, implying ….”.*

11. The meaning of the red lines in Figure 1D should be clarified. Were all scenarios considered? Or does this figure indicate that only 56% of contact scenarios are analyzed?

*We modified the legend of the Figure 1D to clarify that the red lines indicate the two most frequent organizing patterns of P, E1, E2, and, later, E3, according to their relative positions along the genome.*

*In order to demonstrate the hypothesis of 3D structures for ECs more clearly, we included the organization model for the second most frequent scenario (16%) into the Supplemental Figure S4C.*

*Yes, we performed a statistical analysis for all possible scenarios (Figures 1A, 1B, 1C, and Figures 2, 3, and 4 in the Results section), including the two most frequent scenarios (Scenario 1 and 2). In addition, these two scenarios were also used for the proposed models of 3D organization of an enhancer chain (Figure 1E, Supplemental Figure S4C), and these two scenarios account for 56% of all possible configurations.*

12. The analysis of convergent CTCF motifs (p7: 156-165) needs to be clarified. These motifs are between the E1 and P? Shouldn't they be within or on either side of the enhancer?

*Previous studies have suggested that when a loop is formed, the orientation of two CTCF motifs located at different contacting points is usually convergent [Tang Z, et. al. Cell, 163:1611, 2015], therefore we focused on pairs of CTCF motifs located at two different elements of an enhancer chain. In the revised version of the manuscript, we clarified that these are pairs of CTCF motifs located in two different regions that are in contact with each other. By calculating the fraction of promoter-enhancer or enhancer-enhancer pairs with convergent CTCF motifs, we demonstrate the difference in the ability to form chromatin loops for chain enhancers. The P-E1 and E1-E2 pairs show a significantly higher frequency of harboring two convergent CTCF motifs than P-E2, P-E3, and E2-E3 (Figure 2A), suggesting a possible loop formation linking P to E1 and E1 to E2.*

*We added the following statement to the revised manuscript:*

*“We first compared the fraction of any two regulatory elements of an EC (for example, P-E1, PE2, P-E3, E1-E2, and E2-E3) harboring convergent CTCF binding sites in GM12878 cells.”*

13. Are the loop boundaries in 2C all in reference to GM12878 (p7: 174-175)?

*These loop boundaries are associated with four corresponding tissues, not GM12878 only. We clarified this in the Methods section of the revised manuscript:*

*“The boundaries of chromatin loops and sub-TADs were obtained for the matching tissue in each case.”*

14. E1 enhancers in HUVEC cells don't contain a larger fraction of Hi-C contacts across TADs. Why this is? Also, isn't the whole point of defining TADs that there are very few interactions across their boundaries (p7: 177-180)?

*This is a very important point, which requires a clarification on the TAD data usage on our side. There are TADs and sub-TADs (or sub-domains), which distinguish long-range from short-range interactions (Rao et. al. Cell, 159:1665, 2014). We assumed that enhancer chains should not cross TAD boundaries, as this would invalidate enhancer chains as characteristics of a single regulatory domain. We implicitly assumed that enhancer chains might connect different sub-TADs into a single TAD. However, the language used in the original manuscript failed to make this important distinction and we apologize for this oversight on our part.*

*In the revised version of the manuscript, we clarify that we are investigating the role of enhancer chains in connecting sub-TADs within TADs. We show that enhancer chains largely do not cross TAD borders (98% of enhancer chains are located within a single TAD; Supplemental Figure S5A), while they do commonly connect sub-TADs within a TAD (Figure 2D). The connections formed by E1 have the largest impact on sub-TAD bridging and are followed by E2 and E3+ contacts.*

*In the main text, “TADs” have been changed to “sub-TADs” and Figure 2D was updated.*

*Also, we added a clarification statement to the Results section:*

*“However, the enhancer chains largely do not cross TAD borders (98% of enhancer chains are located within a single TAD, Supplemental Figure S5A).”*

*Figure 2D and Supplemental Figure S5A. E1 enhancers maintain a higher fraction of Hi-C interactions across sub-TADs, while EC Hi-C interactions largely remain inside individual TADs.*

15. I think the number of contacts in 3A doesn't necessarily reflect the number of discrete elements that the enhancer connects to; there might be cases of many contacts to the same region or few contacts to a lot of regions that would look identical in this representation. This makes it challenging to interpret this plot.

*We agree with the reviewer’s concern and apologize for the confusion. Figure 3A is actually calculating the number of unique promoters and enhancers connected to a particular enhancer. If a region has multiple contacts to another region, this will be counted as only one contact between the two regions. The y-axis label in Figure 3A has been corrected and we clarified this in the manuscript:*

*“E1s feature a significantly larger number of connected promoters and enhancers than E2s”.*

*Figure 3A. The number of connected promoters and enhancers for different chain enhancers*

6. The tissue specificity plot in 3C doesn't completely convince me that E1 having greater tissue activity similarity is a universal trend. I agree that they all have somewhat similar levels of specificity, but I find the interpretation a little strong. Also, the median Jaccard similarities are quite low. Can the authors comment on this?

*We agree with the reviewer that the overall difference is rather low, although the correlated tissue specificity with a promoter is higher for E1 than other enhancers. In line with the reviewer’s suggestion, we modified the corresponding sentence in the revised manuscript to:*

*“Our results show that for cases when promoters have only one E1, that E1 enhancer has an elevated tissue-specificity similarity to its associated promoters compared to other EC enhancers, although this difference reaches only statistical significance for two out of four tissues (Supplemental Figure S5C). This finding suggests a possible role of ECs, especially E1s, in establishing the baseline tissue-specificity of their target genes.”*

*The original Figure 3C was updated using promoters associated with one EC only and moved to Supplemental materials (Supplemental Figure S5A).*

*Next, in order to address why the overall Jaccard Index is low, we calculated the tissue-specificity of each promoter and its associated E1, represented by the fraction of total tissues in which they are active. As shown in the following plot (Supplemental Figure S5E), the promoters are active in more tissues and thus less tissue specific. In comparison, the chain enhancers, especial E1s, are very tissue specific. This difference in tissue specificity between a promoter and its chain enhancers leads to a small number of common active tissues shared by them, which results in the overall small value of the Jaccard Index.*

*We added the following clarification statement to the Results section 3:*

*“Since the overall Jaccard Index is low, we calculated the tissue-specificity of each promoter and its associated E1 and showed that the promoters are nearly ubiquitously active across tissues and thus less tissue specific. By contrast, the E1 enhancers are very tissue specific (Supplemental*

*Figure S5E). This difference in tissue specificity between promoter and its chain enhancers leads to small number of common active tissues shared by them, which results in the overall small value of the Jaccard Index”.*

*Supplemental Figure S5. C). The tissue specificity similarity between the target promoter and associated chain enhancers. Jaccard Index was used to compare the enhancer and promoter lists of active tissues. E). Tissue specificity of target promoters and their associated E1s.*

17. In the eQTL analysis, given that many of the contexts have eQTL data from the cell line considered (or a similar tissue sample), is there tissue/cell line specificity among the enhancer chains? Does the first enhancer maintain higher density of eQTL for the relevant context?

*Following the reviewer’s suggestion, we selected only the eQTL dataset best matching the tissue specific it of an enhancer chain (“whole blood” for GM12878 and K562, “breast mammary” for HMEC and “artery aorta” for HUVEC) for this analysis. The results did not change qualitatively, and the E1 enhancer still maintained the highest density of tissue-specific eQTLs.*

*The main text, Figure 3E (copied below), and the Methods section 5 were updated to reflect this change: “For tissue-specific variants, “whole blood” eQTLs were selected for GM12878 and K562 enhancer chains, “breast mammary” eQTLs for HMEC, and “artery aorta” eQTLs for HUVEC.”*

*Figure 3. E) The density of expression quantitative trait loci (eQTL) variants for different EC enhancers. “Whole blood” eQTLs were selected for GM12878 and K562 enhancer chains, “breast mammary” eQTLs for HMEC, and “artery aorta” eQTLs for HUVEC.*

18. Does the additive model take into account multiple ECs acting on the same gene?

*To address the effects of multiple ECs targeting the same gene, we split EC target genes into two different cases: associated with multiple ECs (n>=2) vs one EC (revised Figure 4A). The gene expression level associated with one EC is significantly higher than the level associated with one single enhancer in a gene locus (1.7-fold increase, p-value < 0.001). In addition, gene expression levels associated with multiple ECs are significantly higher than the level of one EC (1.9-fold increase, p-value < 0.01).*

*We added the following statement to the Results section:*

*“We found that the presence of an EC elevates the level of gene expression significantly compared to those genes containing only one enhancer in their loci (a 1.7-fold increase, p-value < 0.001, the Wilcoxon rank-sum test), suggesting that multiple enhancers within an EC may boost the expression of the target gene. A similar trend is observed when comparing the expression level of genes associated with multiple ECs to that of genes with one EC (a 1.9-fold increase, p value < 0.01, the Wilcoxon rank-sum test).”*

*Figure 4A. The level of gene expression associated with multiple ECs (labeled “multi ECs”), one EC only, one enhancer in a gene locus only (“one enh”), and an EC connecting to the target promoter through a non-active-enhancer (“non-enh”) DNA fragment, respectively.*

**Reviewer 2**

* 1. The language used in the introduction pertaining to the existence and function of enhancer hubs is too strong. Indeed, there is little direct evidence of true enhancer hubs whose activity/interactions are more than the accumulation of their individual activities/interactions.

*As specified by the reviewer, we carefully went through the main text and either removed or modified statements pertaining to hub enhancers in order to avoid unnecessary confusion about the true nature of these enhancers. The term “hub” enhancers is no longer used in the revised version of the manuscript.*

*For example:*

*1). We changed the sentence “… the hierarchical structure of enhancer networks, and the hub structure of open chromatin interactions inside SEs” to: “… the hierarchical structure of enhancer networks, and open chromatin interactions inside SEs”*

*2). We changed the sentence “…including a spatial enhancer hub formed by interacting enhancers within the beta-globin SE” to “…including a group of interacting enhancers within the beta-globin SE”*

* The biggest concerns result from the definition of enhancer chains.

o First, there are several attributes of ECs that are poorly described/characterized:

§ 2. If an enhancer connects to multiple ECs and is chosen for one based on its closest rank, are the other connections completely ignored, and/or does this affect the rank of this enhancer relative to the non-chosen ECs?

*We rank enhancers according to their rank across all ECs (the smallest number of consecutive Hi-C interactions with the closest promoters). For example, if an enhancer is ranked second (E2) and third (E3) in two different ECs, it will be treated as an E2 enhancer in the corresponding EC only. To avoid duplication, it will not be double-counted as an E3 in the second EC. As shown in the Supplemental Figure S1C-D, different ECs may share common enhancers at the same rank and one enhancer cannot be assigned to two different ranks. Using this definition, all connected enhancers in the network are assigned to at least one EC. We updated the definition of ECs in Supplemental Figure S1 and added a clarification statement “We used the shortest distance approach in the construction of ECs. Namely, each enhancer was connected to the promoter using the smallest number of intermediate contacting enhancers possible. For example, in the case of a shorter and a longer path (measured as the number of contacting enhancers), only the shorter path was selected (Supplemental Figure S1C). In the case of two equidistant paths, both were selected (Supplemental Figure S1D). However, to avoid bias and double-counting, enhancers from equidistant paths were used only once in our statistical tests.” to the Methods section of the revised manuscript.*

*Supplemental Figure S1. C) In the case of a shorter (solid line) and a longer (dashed line) path (measured as a number of intermediate contacting enhancers), only the shorter path was selected. D) Different ECs may share common enhancers from the same rank.*

§ 3. How are chains defined if an enhancer is the E1 for multiple promoters?

*A chain with an E1 shared by two promoters was considered a single EC and was not doublecounted.*

*In this case, both promoters were included into P-E1 related analyses. There were 2,055 cases with ECs ending at two promoters (23% of all ECs). To investigate the influence of this set of ECs on our results, we split all the ECs into two separate sets: an E1 associated with only one promoter (1P-E1) and an E1 associated with multiple promoters (MP-E1). We compared the major genomic features between these two EC sets. The distance to the target promoter and the number of connected regulatory elements maintain similar trends between the two sets and are in agreement with the patterns for all ECs (new Supplemental Figure S6A and S6B). However, the E1 that connects to multiple promoters has significantly more tissue-specific eQTLs than the E1 connecting to only one promoter, suggesting the critical role of E1s in regulating multiple gene expression (new Supplemental Figure S6C). In addition, although the multiple genes with their promoters connected to the same E1 are more likely to be co-expressed (revised Figure 4C), their overall level of expression is lower than the 1P-E1 genes (new Supplemental Figure S6D).*

*We added a clarification statement to the revised manuscript in Results section 4:*

*“However, about 23% of all ECs are shared by more than one promoter, in which an enhancer is the E1 for multiple promoters. The fraction of EC enhancers associated with multiple promoters among all enhancers connected in the regulatory element networks is 0.39, 0.13, 0.12 and 0.39, in GM12878, HMEC, HUVEC and K562, respectively. To investigate the influence of this set of ECs on our results above, we partitioned all ECs into two separate sets: E1s associated with only one promoter (1P-E1) and E1s associated with multiple promoters (MP-E1). We compared the major genomic features between these two EC sets. The distance to the target promoter and the number of connected regulatory elements maintain similar trends between the two sets and are in agreement with the patterns for all ECs (Supplemental Figure S6A and S6B). Interestingly, the E1s that connect to multiple promoters have significantly more tissue-specific eQTLs than the E1 connecting to only one promoter, suggesting their critical role in regulating multiple genes (Supplemental Figure S6C). Notably, the expression levels of the genes sharing ECs are lower than those of the genes with only a single EC (Supplemental Figure S6D), although the multiple genes contacting the same EC are more likely to be co-expressed (Figure 4C), suggesting that the existence of competing target genes linked to the same EC might partially mitigate the expression of each gene.”*

*Supplemental Figure S6. Comparing the genomic features between ECs with E1 associated with only one promoter (1P-E1) and with multiple promoters (MP-E1). The distance to the target promoter (A), the number of connected promoters/enhancers (B), density of tissue-specific eQTLs (C) and associated gene expression levels (D).*

§ 4. How often are enhancers part of ECs with multiple promoters before rank-based choices?

*The fraction of enhancers in an EC associated with multiple promoters among all connected enhancers in the regulatory element networks before the application of single ranking was 0.39, 0.13, 0.12, and 0.39, in GM12878, HMEC, HUVEC and K562, respectively. After distance based ranking, these enhancers became categorized as EC enhancers. We added a clarification statement to the Results section 4 of the revised manuscript:*

*“The fraction of enhancers in an EC associated with multiple promoters among all connected enhancers in the regulatory element networks before the application of single ranking was 0.39, 0.13, 0.12, and 0.39 in GM12878, HMEC, HUVEC and K562, respectively. After single ranking, these enhancers became categorized as EC enhancers.”*

§ 5. Lines 107-108. What is the distribution of the number of E1s per promoter?+

*The plot below shows the distribution of the number of E1s per promoter (new Supplemental Figure S3D). The majority of promoters contact a single E1. Please see our response above for an detailed comparison of promoters contacting a single E1 versus multiple E1s.*

*Supplemental Figure S3D. The overall distribution of E1s per promoter combined for four tissues.*

§ 6. How do you define a distinct EC? Is it different E1s, or any different combination of participating enhancers or any different ordering? Given the example EC in Supplemental Fig. 1A, one can draw many different paths that connect consecutive enhancer ranks. Are these all considered different ECs?

*As stated in the response to the comment #2 of the reviewer, we used the shortest distance approach in construction of ECs. Namely, each enhancer was connected to the promoter using the smallest number of intermediate contacting enhancers possible. In the case of the Supplemental Figure S1E referenced by the reviewer, there were eight possible ECs and we are color coding all of them in the revised version of this Figure.*

*Supplemental Figure S1. Examples of enhancer chains (ECs). A) One sample of the regulatory element networks. B) An EC associated with a promoter (P) is defined as a set of contiguously connected enhancers (at least two) through enhancer-enhancer Hi-C interactions, according to their ranks associated to this promoter. The first enhancer in a chain that contacts the promoter directly is called step-one enhancer (E1), the second one in the chain is called step-two enhancer (E2), and so on. C) In the case of a shorter (solid line) and a longer (dashed line) path (measured as a number of intermediate contacting enhancers), only the shorter path was selected. D) Different ECs may share common enhancers from the same rank. E) Theoretical schematic of enhancer chain overlaps. Eight different ECs defined according to the rank of connected enhancers relative to their closest target promoter. These ECs may share common enhancers from the same rank. At least one enhancer should be different to distinguish between two different ECs. The closest rank of an EC enhancer to the promoter is used to describe the enhancer.*

o 7. Secondly, is there any reason to believe that a chain-based, single path analysis approach is the correct or preferable approach? This is especially important in cases where a given promoter has multiple direct "E1" contacts. What is the motivation to consider chains as separate units?

*The key reasoning behind establishing enhancer chains was to compute the shortest distance between an enhancer and its target promoter(s) and to highlight the indirect nature of enhancer- promoter interactions in cases when an enhancer is separated from its target promoter by one or more intermediate enhancers. To further investigate the bifurcation of enhancer chains, we quantified the number of contacts between enhancers from different enhancer chains in each original regulatory network and a control set of same network but with enhancer IDs shuffled for 10 times, across all four tissues. We found that about 25% of non-overlapped ECs associated with the same promoter have interactions with each other, compared to 77% in the control set, on average (p-value < 2.2´10-16 , the Wilcoxon rank sum test). Only 18% of the EC enhancers pairs with the same rank but from different ECs display inter-EC interactions, compared to 43% in the control set (p-value < 2.2´10- 16, the Wilcoxon rank sum test) (Supplemental Figure S4B). (We only considered interactions between EC enhancers with the same rank, otherwise new* *chains could have been identified through the bridge of En-Em interactions between different ECs based on the definition of EC.) These inter-EC enhancer pairs also overlap both anchors of a loop with a significantly lower frequency than EC enhancer pairs (E1-E2, E2-E3, and so on, Supplemental Figure S4C), suggesting that few chromatin loops formed between them. These relatively limited contacts between different ECs advocate for the presence of well-defined ECs and justify the selection of ECs as a backbone of our study.*

*We added a clarification statement to the Results section:*

*“The key reasoning behind establishing ECs was to compute the shortest distance between an enhancer and its target promoter(s) and to highlight the indirect nature of enhancer-promoter interactions in cases when an enhancer is separated from its target promoter by one or more intermediate enhancers. To further investigate the bifurcation of enhancer chains, we quantified the number of contacts between enhancers from different enhancer chains in each original regulatory network and a control set of same network but with enhancer IDs shuffled for 10 times, across all four tissues. We found that, on average, about 25% of non-overlapped ECs associated with same promoter have interactions with each other, compared to 77% in the control set (p value< 2.2´10- 16 , the Wilcoxon rank sum test). Only 18% of the EC enhancers pairs with the same rank but from different ECs display inter-EC interactions, compared to 43% in the control set (p-value < 2.2´10-16, the Wilcoxon rank sum test) (Supplemental Figure S4B). These enhancer pairs also overlap both anchors of a loop with a significantly lower frequency than EC enhancer pairs (E1-E2, E2-E3, and so on), suggesting that few chromatin loops formed between them (Supplemental Figure S4C). These relatively limited contacts between different ECs advocate for the presence of well-defined ECs and justify the selection of ECs as a backbone of our study.”*

*Supplemental Figure S4. B) Among the non-overlapped ECs, the fraction of ECs that maintain inter-chain Hi-C interactions and the fraction of enhancer pairs from two different ECs connected by Hi-C interactions across four tissues. C) Fraction of enhancer pairs from the same (intra-) and different (inter-) ECs overlap two boundaries of a loop simultaneously.*

* 8. Why look at the statistics of enhancers being upstream or downstream of promoters? Is there any evidence that this relative positioning in the linear genome is somehow important?

*It has been reported that the first intron of a gene locus is very important in gene regulation and might contain regulatory elements such as enhancers and silencers (Rossi, P. and de Crombrugghe, B. PNAS,84:5590, 1987; Scohy, S. et. al. NAR, 28:3743, 2000). To investigate whether this positional preference exists in the promoters associated with ECs, we looked at the cases of EC enhancers being upstream and downstream of promoters. However, we didn’t find a significant difference between these two cases (53% of E1s and 55% of E2s locate downstream of the target promoter). To avoid confusion, we modified Figure 1E and Supplemental Figure S4A by removing the direction of gene transcription.*

*We added a clarification statement in Results section 1 of the revised manuscript:*

*“Since alternation of the relative positions of the promoters and enhancers may lead to different 3D structures (Figure 1D) and the first intron of a gene has been reported to be an important component of gene regulation [35, 36], , we compared the cases in which EC enhancers are located upstream or downstream of promoters to investigate whether this positional preference exists in the ECs. However, we did not find a significant bias towards either of the two situations.*

*Regardless of either the upstream or downstream position of the EC enhancers with regard to the promoters, we proposed two organization patterns for the top two most frequent situations (40% and 16% of the total cases), called Scenarios 1 and 2, respectively, in which E1 and E2 are in the same and different sides of a promoter (Figure 1E and Supplemental Figure S4C).”*

* 9. Lines 136-141. These are hypotheses, quite far from evidence-based facts, and should therefore be stated as such.

*We agree with the reviewer and modified this statement to highlight the speculative nature of it: “Based on the most frequent scenarios, we proposed a hypothesis of how an EC is built in 3D space: 1) the E1 is …”*

* 10. Is Fig 2a not mostly the result of your definition of E2s, which do not have evidence of direct contact with the promoter and therefore shouldn't show convergent ctcf motifs?

*We agree with the reviewer that there should be no direct contacts between E2 and promoters, and as a consequence there should be few if any convergent CTCF motif pairs between them, but only if our assumption or the way to define the structure of enhancer chains is correct. In effect, the analysis presented in Figure 2A validates our hypothesis that there is no direct interaction between E2 and P and their spatial contact is established through an intermediate E1. Not only that, but also the Figure 2A results also demonstrate a formation of two CTCF-anchored loops, one connecting P with E1 and another one connecting E1 with E2 during a formation of an indirect E2-P contact. We added a clarification statement to the Results section 2: “Since the convergent CTCF motifs are crucial for the formation of chromatin loops, this observation validates our hypothesis that there is no direct interactions between an E2 and a P and that their spatial contact is established through an intermediate E1. It also demonstrates a formation of two CTCF-anchored loops, one connecting P with E1 and another one connecting E1 with E2 during the formation of an indirect E2-P contact.”*

* 11. Are E1s different in terms of fragment length and/or GC content? Presumably these known technical artifacts for Hi-C would be normalized out with ICE, but this is a necessary sanity check.

*The reviewer is absolutely correct. These biases are largely balanced out by ICE. However, to further address these potential biases, we performed an additional analysis of fragment length and GC content in the Hi-C data mapped to EC. Since the bias of distance between restriction sites or fragment length may lead to the underrepresentation of very short- or very long-range interactions (Yaffe and Tanay, Nat. Genet. 43:1059, 2011), we compared the distance between EC enhancers and the genomic regions with which they were in contact. Although E1s show a significantly larger range of contacts than other chain enhancers, the actual median values are very close among all En categories (Supplemental Figure S2A). The average length of interactions for E1s is only 1.1-fold higher than that for E2s. This observed difference in range of interactions is not sufficient to explain the effects observed by this study (p-value < 0.001, the Wilcoxon rank sum test). One explanation for this difference might be the intrinsic ability of E1s to form distal interactions (Figure 2C and 2D). In addition, the interactions for P-E1 and E1-E2, E2-E3… are all in the middle distances, mostly shorter than 1 Mb, so the influence of fragment length should be limited for EC enhancers. Similarly, the median value of GC content is similar among all En categories in the four tissues (Supplemental Figure S2B). These results indicate that the Hi-C biases are not the primary factors in differentiating E1s from other chain enhancers.*

*The Supplemental Figure S2 has been added to the revised version of the manuscript and the following statement has been added to the Methods section 3:*

*“In order to evaluate the influence of possible biases from Hi-C experiments on our results, we performed a comparison among EC enhancers to identify the potential biases caused by the fragment length and GC content in Hi-C experiments. Since the bias in distance between restriction sites or fragment length may lead to the underrepresentation of very short or very long range interactions [46], we compared the distance between EC enhancers and the genomic regions with which they were in contact. Although E1s show a significantly larger range of contacts than other chain enhancers, the actual median values are very close among all En categories (Supplemental Figure S2A). The average length of interactions for E1s is only 1.1-fold higher than that for E2s. This observed difference in range of interactions is not sufficient to explain the effects observed by this study (p-value < 0.001, the Wilcoxon rank sum test). One explanation for this difference might be the intrinsic ability of E1s to form distal interactions (Figure 2C and 2D). In addition, the interactions for P-E1 and E1-E2, E2-E3… are all in the middle distances, mostly shorter than 1 Mb, so the influence of fragment length should be limited for EC enhancers.*

*Similarly, the median value of GC content is similar among all En categories in the four tissues (Supplemental Figure S2B). These results indicate that the Hi-C biases are not the primary factor in differentiating E1s from other chain enhancers.”*

*Supplemental Figure S2. Distance of Hi-C interactions (A) and GC content (B) for EC enhancers across four tissues.*

* 12. Line 189, how can the average number of promoter-E1 contacts be less than 1? I thought an enhancer could only be an E1 if it directly contacts a promoter?

*In cases when there are multiple E1s connected to a single promoter, we counted a fractional unique promoter for those contacts and that led to the average count of promoters connected to E1 being less than 1. Considering the reviewer’s question, we realize that our approach to counting promoter-enhancer contacts might have been confusing. Therefore, we changed our approach to an E1-centric approach and counted full promoters, even if they are contacted by multiple E1s. The revised average number of promoters contacting E1 subsequently changed to 1.4.*

* 13. Line 191, how can E1s have such large BC scores when in the methods it states that E1 BC scores are set to zero?

*We agree with the reviewer’s concern and apologize for the confusion. We defined zero BC scores only for promoters connected to a single enhancer. The corrected statement is updated in Methods section in the revised manuscript:*

*“For the cases when a promoter is connected to a single enhancer only, the BC score is set to zero.”*

* 14. Line 194. Maybe this suggests that E1s are *constituents* of hub enhancers, but, as I understand your definition, an E1 is one enhancer and therefore can't be a hub by itself.

*Yes, the reviewer is absolutely correct that a hub, by definition, should be connected to multiple enhancers. To clarify, we thus modified the statement to: “… indicates the ability of E1s to connect to multiple enhancers”.*

* 15. Line 198-199, This is only worth considering if the promoter has only one E1, otherwise

it is misleading?

*The reviewer is correct. In the 23% of cases, when a promoter is in contact with multiple E1, there are confounding effects of multiple ECs regulating one gene. To exclude the impact of multiple ECs, we limited the analysis to the cases when a promoter is connected to a single E1 only (new Supplemental Figure S5C; copied below). Our results didn’t change qualitatively upon this subselection of promoters, and the conclusions on the tissue-specificity remained the same. To explore this aspect of the study further, we contrasted this analysis to the cases when a promoter is in a contact with multiple ECs (new Supplemental Figure S5D). In the latter case, the trend effectively disappears, suggesting a more complex regulatory program in loci with multiple Ecs regulating the same gene.*

*We added a clarification statement to the Results section:*

*“Our results show that for the cases when promoters have only one E1, that E1 enhancer has an elevated tissue specificity similarity to its associated promoters compared to other EC enhancers, although this difference only reaches statistical significance for two out of four tissues (Supplemental Figure S5C). This finding suggests a possible role of ECs, especially E1s, in establishing the baseline tissue-specificity of their target genes. However, in the case when a promoter is in contact with multiple ECs, the trend effectively disappears (new Supplemental Figure S5D), suggesting a more complex regulatory paradigm in loci with multiple ECs regulating the same gene.”*

*Figure 3C was moved to Supplemental materials (Supplemental Figure S5C).*

*Supplemental Figure S5. The tissue specificity similarity between the target promoter and the associated chain enhancers for promoters connected to a single E1 (C) and to multiple E1s (D).*

* 16. Exactly what is being measured for the Jaccard index in figure 3c?

*The original Figure 3C has been moved to revised Supplemental Figure S5C in the manuscript. We calculated the tissue-specificity similarity between the promoter and each of the enhancers along an EC. We measured the activity using H3K27ac peaks and computed the fraction of tissues, in which a pair of elements is active. For example, for each pair of P-E1, we calculated the number of tissues (out of 20 total), in which both P and E1 have H3K27ac peaks. After that, we compared the similarity of these two lists using the Jaccard Index to demonstrate the tissue specificity similarity of each P-E1 pair. Similar procedures were applied to P-E2 and P-E3 pairs.*

*We added these clarifying statements to the Results section 3:*

*“To verify this, we calculated the tissue-specificity similarity between the promoter and each of the enhancers along an EC. We measured their co-activities using H3K27ac peaks and computed the fraction of tissues in which each element in a pair is active.”*

*And Methods section 2:*

*“We would like to investigate if E1 is more frequently associated with underpinning the regulatory program of its target gene than rest of the EC enhancers by calculating the tissue-specificity similarity between the promoter and each of the enhancers along the chain. We measured their co-activities using H3K27ac peaks and computed the fraction of tissues in which a pair of elements is active. For example, for each pair of P-E1, we calculated the number of tissues (out of 20 total) in which both P and E1 overlap H3K27ac peaks. After that, we compared the similarity of these two lists using the Jaccard Index to demonstrate the tissue-specificity similarity of each P-E1 pair. Similar procedures were applied to P-E2 and P-E3 pairs.”*

* 17. It is not so clear how much more similar E1 is to promoters compared to others (Figure 3d). It seems what is presented is not actually a direct comparison between the two.

*To facilitate the interpretation of the results, we focused on the fold-enrichment of the same top 30 TFBSs specific to the promoters of genes regulated by ECs in GM12878 and K562 cell lines. In the revised version of the manuscript, the aggregate fold enrichment is presented as distributions binned by the element class (promoters (P), E1, E2, and E3+ enhancers) (Figure 3C copied below). This comparison clearly demonstrates that the fold enrichment distribution has a significantly lower median value for E2 and E3 enhancers as compared with E1 enhancers and promoters.*

*Please note that the original Figure 3D has been relabeled as Figure 3C (while the original Figure 3C has been moved to Supplemental materials).*

*Figure 3C. The fold-enrichment of the top 30 TFBSs specific to promoters regulated by ECs in GM12878 and K562 cell lines (profiled across promoters (P), E1, E2, and E3+ enhancers). * - pvalue < 0.05, ** - p-value < 0.001, p-values are calculated using the Wilcoxon rank sum test.*

* 18. Figure 3e, these are very low numbers, and it is unclear that they mean anything.

*We understand the reviewer’s concern and to better evaluate the significance of these results, we calculated the p-value of these fractions using randomly selected enhancers as a control set (we randomly selected enhancers with a total number and distance to the target promoters matching the original E1, E2, and E3 categories, respectively, in the corresponding tissue). We observed that 1) chain enhancers tend to maintain their relative position in an EC (E1-E1 in red and E2-E2 in blue) and 2) E1 is less tissue specific, as it shows the larger total fraction of EC position preservation across four different tissues. The p-values were added to the Results section and Figure 3D in the revised manuscript, and the corresponding section reads now as: “For those chained enhancers in one tissue, E1s most likely remain as E1s (9.5%, p-value < 10- 10 , the Binomial test) instead of changing to either E2s (3.4%) or E3s in another tissue (1.6%, pvalue < 0.001, the Binomial test; disregarding cases in which an enhancer does not overlap an H3K27ac mark or is not part of a chain). Similarly, E2s in one tissue tend to remain E2s (6.1%, pvalue < 10- 10 , the Binomial test) again in a different tissue rather than changing to either E1s (2.8%) or E3s (1.0%).” Please note that the original Figure 3E was relabeled as Figure 3D in the revised manuscript.*

*Figure 3D. The fraction of EC enhancers that either maintain or switch their positions in an EC across four tissues. N1, N2, and N3 are the total number of enhancers for each category of enhancers combined across four tissues.*

* 19. Line 233-236, this does not indicate an additive function.

*We removed the statement of additive function from the corresponding sentence (“…indicating an additive function of multiple enhancers from an EC in regulating the target gene” on line 235- 236 was deleted).*

* 20. Figure 4. It seems possible that single and/or non-chain enhancers used for comparisons in this figure could just be false-positive enhancer candidates. Is there any strong evidence to consider these single or non-chains regions as enhancers at all? This question could also be posed for E1 versus E2,3,4… enhancers. Could it be that E1 enhancers are true enhancers and the others just be false positives?

*Please see our response to comment #6 of the first Reviewer about active open state, in which we addressed the likelihood of E2 and E3+ being false positive enhancer predictions. To test whether single and non-chain enhancers are false positive predictions or not, we added this category to the comparison and the results are presented in the Supplemental Figure S8 below.*

*In particular, the mutations in E1s have the most pronounced impact on the level of target gene expression according to the eQTL data profiled across four cell types. The eQTL density in all categories of enhancers is non-negligible and the eQTL densities in E2 and E3+ chain enhancers decrease with the degree of separation from the promoter. In addition, the fold-enrichment of bound TFs according to ChIP-seq data for E1, E2, E3, and single and non-chain enhancers is at a similar level that, in turn, is significantly higher than that in non-enhancer regions of open chromatin, suggesting that all enhancers in an EC, as well as the single and non-chain enhancers,*

*are actively bound by TFs and are functionally correlated with the expression level of their target genes (Supplemental Figure S8C and S8D). This analysis rejects a hypothesis of E2 and E3+ enhancers being false positives.*

*These plots were added as the Supplemental Figure S8 and the following statement was added to the Results section 4:*

*“Finally, to test the hypothesis that non-E1 enhancers are irrelevant to gene regulation and simply represent open chromatin regions or false positive enhancer predictions, we compared the density of tissue-specific eQTLs in E1, non-E1, and single and non-chain enhancers (Supplemental Figure S8). The mutations in E1s have the most pronounced impact on the level of target gene expression according to the eQTL data profiled across four cell types. The eQTL density in all categories of enhancers is non-negligible and the density in E2 and E3+ chain enhancers decreases with the degree of separation from the promoter. In addition, the foldenrichment of bound TFs according to ChIP-seq data for E1, E2, E3, and single and non-chain enhancers is at a similar level (which, in turn, is significantly higher than that in non-enhancer regions of open chromatin), suggesting that all enhancers in an EC, as well as the single and nonchain enhancers, are actively bound by TFs and contribute to the expression level of their target genes (Supplemental Figure S8C and S8D).”*

*Supplemental Figure S8. C) The density of expression quantitative trait loci (eQTL) variants for EC and single and non-chain enhancers as well as non-enhancer regions of open chromatin. D) The fold enrichment of TFBSs for different enhancer categories using all open chromatin regions (DNase I ChIP-seq peaks) as control, in GM12878 and K562 cell lines, respectively. ** - p-value < 0.001, *** - p-value < 1´10-10, p-values are calculated using the Wilcoxon rank sum test.*

**Second round of review**

**Reviewer 1**

I thank the authors for their thorough and extensive responses to my comments and revisions to the manuscript. I feel that it's clarity and support for conclusions are improved.

While I feel that some of the interpretations of mainly correlative analyses may still be too strong, I nevertheless believe that the manuscript presents many novel and thought-provoking analyses that will help move our understanding of enhancer interactions forward. 

I have just a few minor suggestions of clarifications to the Abstract:

1.     Line 23: Change: "indicative" to "suggestive"
2.     Line 24: Without detailed knowledge of the methods employed it is immediately counterintuitive how the first element in an enhancer chain could be a "neutral DNA element" rather than an enhancer. I suggest that the authors clarify the wording here to avoid this potential confusion.
3.     The Conclusion statement should be more specific to the actual results of this paper and not rely on the authors' perception of what has been "overlooked." Furthermore, I don't think it would come as a surprise to many that distal enhancers can influence complex regulatory programs.

**Reviewer 2**

The study by Song et al. has shown significant improvement. Overall, the EC approach is better supported and the definitions are clearer than the initial submission, and new analyses performed in response to reviewer concerns have proven useful. Especially appreciated were the shuffling experiment and subsequent analyses the authors took to validate the EC approach, the comparisons with single and non-chain enhancers, and the observations on ECs contacting multiple promoters. Together, these analyses seem critical to convince and inform the reader as to the basic characteristics of ECs compared to other regulatory elements. One remaining suggestion, therefore, is to reorganize the manuscript such that some comparisons between ECs and single, non-chain, shuffled ECs, and multiple-promoter ECs are highlighted as main figure panels toward the beginning of the study (as opposed to late supplemental figures), thereby convincingly establishing the relative importance of ECs early in the text. This will allow the reader to more thoroughly dissect the detailed characteristics of ECs and the apparent differences between enhancer ranks within ECs. Otherwise, in the current version of the text one is too frequently returning to re-evaluate initial figures in light of findings from subsequent ones.
The second remaining criticism is that it seems the authors are under-emphasizing one of the more striking aspects of their analysis: that E2+ enhancers are important. While the influence and characteristics of E1 enhancers is very clear and necessary to establish as the authors have done convincingly (especially in contrast to single and non-chain enhancers), a devil's advocate could argue that this is not entirely surprising given that they are in direct contact with promoters by definition. What seems possibly even more important are the observations the authors have made on E2+ enhancers, which share some characteristics with E1 and single enhancers, but without directly contacting promoters. Therefore, the manuscript might benefit from a more balanced presentation of the EC as a whole, rather than the E1-centric perspective the authors have chosen. After all, it is the existence of E2+ enhancers, as the authors have defined them, which convincingly distinguishes ECs from single enhancers.

**Authors Response**

**Point-by-point responses to the reviewers’ comments:**

*We are grateful to both Reviewers for their helpful suggestions. Please find below our point-bypoint response to the remaining*

Reviewers’ concerns.

Reviewer #1:

1. Line 23: Change: "indicative" to "suggestive"

*Done.*

2. Line 24: Without detailed knowledge of the methods employed it is immediately counterintuitive how the first element in an enhancer chain could be a "neutral DNA element" rather than an enhancer. I suggest that the authors clarify the wording here to avoid this potential confusion.

*We modified the corresponding statement, which now reads “However, a chain of enhancers which connects to the target promoter through a neutral DNA segment instead of an enhancer is associated with a significant decrease in target gene expression”.*

3. The Conclusion statement should be more specific to the actual results of this paper and not rely on the authors' perception of what has been "overlooked." Furthermore, I don't think it would come as a surprise to many that distal enhancers can influence complex regulatory programs.

*We modified the conclusion statement in the Abstract, which now reads “The widespread chained structure of gene enhancers in humans reveals that the primary, critical enhancer is distal, commonly located further away than other enhancers. This first, distal enhancer establishes contacts with multiple regulatory elements and safeguards a complex regulatory program of its target gene.”*

Reviewer #2:

1. One remaining suggestion, therefore, is to reorganize the manuscript such that some comparisons between ECs and single, non-chain, shuffled ECs, and multiple-promoter ECs are highlighted as main figure panels toward the beginning of the study (as opposed to late supplemental figures), thereby convincingly establishing the relative importance of ECs early in the text.

*We moved 4 panels of the original Fig. S6 in to the main Figure 4. The revised Figure 4 describes the comparison between ECs and other regulatory elements (non-chain, shuffled ECs, and multiple-promoter ECs) in greater detail.*

2. The second remaining criticism is that it seems the authors are under-emphasizing one of the more striking aspects of their analysis: that E2+ enhancers are important.

*We extended the Discussion section to emphasize the essential contribution from E2+ to gene regulation. The corresponding statement reads as “Although our results emphasize the important and distinguishing characteristics and influence of E1s on target gene expression in contrast to other regulatory elements, such as single and non-chain enhancers, we also observe a significant contribution of E2+ enhancers, which have no direct interactions with the promoter, to the target gene expression. As part of ECs, E2+ enhancers cooperate with E1s to orchestrate gene regulation in a complex manner. It is also likely that these E2+ enhancers are stabilizing the regulatory contact domain dynamically, thereby fine-tuning and amplifying the target gene expression in a cell-specific manner.*
